# Recent Progress in Semitransparent Organic Solar Cells: Photoabsorbent Materials and Design Strategies

**DOI:** 10.3390/mi15040493

**Published:** 2024-04-02

**Authors:** Shabaz Alam, Suhui Sim, Meng Qiang Li, Bong-Jun Chang, Jaewon Lee

**Affiliations:** 1 Department of Chemical Engineering and Applied Chemistry, Chungnam National University, Daejeon 34134, Republic of Korea; shabaz@cnu.ac.kr (S.A.); ssh822@o.cnu.ac.kr (S.S.); 202150699@o.cnu.ac.kr (M.Q.L.); 2 Interface Materials and Chemical Engineering Research Center, Advanced Materials Division, Korea Research Institute of Chemical Technology (KRICT), 141 Gajeongro, Yuseong, Daejeon 34114, Republic of Korea; changbj@krict.re.kr

**Keywords:** semitransparent organic solar cells, non-fullerene acceptors, light utilization efficiency, narrow bandgap

## Abstract

The increasing energy demands of the global community can be met with solar energy. Solution-processed organic solar cells have seen great progress in power conversion efficiencies (PCEs). Semitransparent organic solar cells (ST-OSCs) have made enormous progress in recent years and have been considered one of the most promising solar cell technologies for applications in building-integrated windows, agricultural greenhouses, and wearable energy resources. Therefore, through the synergistic efforts of transparent electrodes, engineering in near-infrared photoabsorbent materials, and device engineering, high-performance ST-OSCs have developed, and PCE and average visible transmittance reach over 10% and 40%, respectively. In this review, we present the recent progress in photoabsorbent material engineering and strategies for enhancing the performance of ST-OSCs to help researchers gain a better understanding of structure–property–performance relationships. To conclude, new design concepts in material engineering and outlook are proposed to facilitate the further development of high-performance ST-OSCs.

## 1. Introduction

Sustainable growth and socioeconomic development heavily depend on energy. At present, carbon emissions resulting from the combustion of fossil fuels pose a significant environmental threat. A key challenge of the 21st century is reducing carbon emissions to net zero in order to slow the effects of environmental change. Renewable energy plays a crucial role in mitigating the impact of climate change. For the production of electricity from renewable resources, utilizing photovoltaic (PV) technologies is one of the most suitable strategies [1,2,3]. The conversion of solar energy into electricity is highly reliant on semiconducting materials, which serve as the central core component within solar cells. The pursuit of semiconducting materials that are eco-friendly, inexpensive, and facile to fabricate has been a longstanding goal of interest for both industry and the academic community involved in PV research [4,5]. Organic solar cells (OSCs) have the potential to serve as low-cost and easily processable materials that result in lower energy payback times [6,7]. State-of-the-art OSCs are typically assembled by employing a solution-processed bulk heterojunction (BHJ) technique, combining donor–acceptor material to create a bi-continuous interpenetrating network and forming a significant interfacial area to facilitate exciton dissociation into free charge carriers [8,9,10,11,12]. The field of OSCs is currently undergoing a second golden age, driven by the emergence of innovative non-fullerene acceptor (NFA) materials and device design, which led to a power conversion efficiency (PCE) of over 19% [13,14]. Recently, a number of stable OSCs with expected lifetimes over 10 years have been documented [15]. 

**Figure 1:**
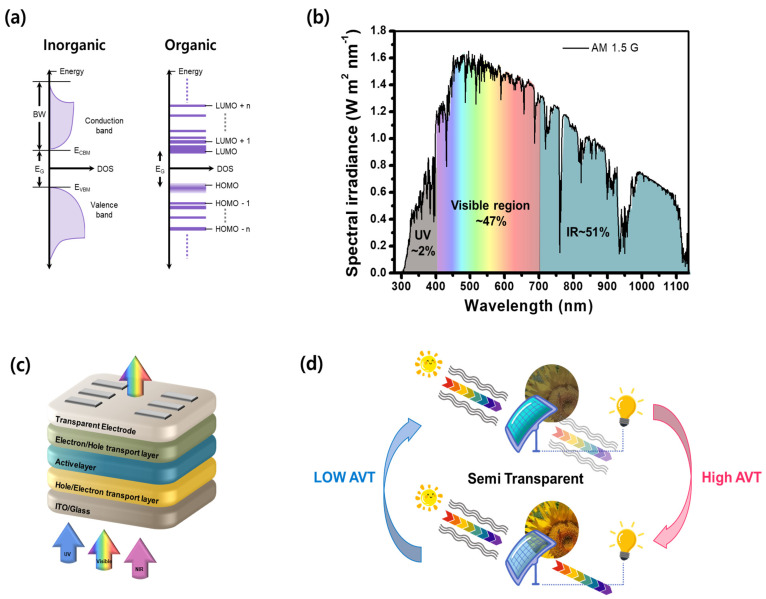
(**a**) Pictorial illustration of the energy level of inorganic and organic semiconductors; (**b**) solar emission spectrum and the energy fraction in the UV, visible, and infrared regions; (**c**) illustration of a visibly transparent (UV−NIR-selective) ST-OSC device; and (**d**) schematic illustration of an ST-OSC module with low and high average visible transmittance (AVT).

All organic semiconductors form Frenkel excitons owing to their low dielectric constant (*ε* ≈ 2–4) and high exciton binding energy [16]. In contrast, inorganic semiconductors form Wannier–Mott excitons contained in covalent bonding with a high dielectric constant (e.g., *ε* ~12 for silicon) and low exciton binding energy, causing electrons to become delocalized across the solid. Owing to this, charges can travel long distances with minimal hindrance, resulting in high mobility and wide energy bands. Due to their broad energy bands, inorganic semiconductor materials are opaque over a wide range of energies and difficult to form transparent solar cells (Figure 1a). Moreover, silicon only acquires an absorption coefficient of approximately 10^2^ cm^−1^ and industrial thickness exceeding 100 μm, making it opaque to visible light. On the other hand, conjugated organic materials have confined band absorption and hold very immense oscillator strength associated with the primary transition, making them strongly absorptive within relatively limited spectral ranges [17]. High absorption coefficients of around 10^5^–10^6^ cm^−1^ and selectively engineered organic materials for the absorption of the solar spectrum in the ultraviolet and near-infrared (NIR) regions while enabling transparency in the visible region made it an ideal candidate to be used in semitransparent (ST) OSCs (Figure 1b–d) [18]. Furthermore, the OSCs do not contain undesirable elements like lead (Pb), broadening their potential applications in building-integrated photovoltaics (BIPVs), self-powered wearable electronic devices, automobiles, remote sensors, agricultural greenhouses, and so on in comparison to thin-film solar cells that do contain lead. 

The primary challenges in realizing efficient ST-OSCs depend on simultaneously achieving high transmittance in the visible region to meet the demand for various applications while also effectively harnessing photons in the UV and NIR regions to maintain high efficiency. A comprehensive molecular design, device physics, and transparent electrode design approach are all crucial to accomplishing this task. The photovoltaic community has only recently dedicated attention to semitransparent OSCs (ST-OSCs). The basic concept behind the development of ST-OSCs is to pass a substantial fraction of the incident solar spectrum within the visible range, which is perceptible to the human eye [19]. The fabrication techniques for ST-OSCs and opaque OSCs are highly similar, allowing progress in traditional OSCs to be easily applied in ST-OSCs and vice versa. Since there is a compromise between light transmittance and efficiency, one major challenge in achieving efficient ST-OSCs is satisfying the requirement for both highly transparent incident light in the visible region and sufficiently harvesting the UV and/or NIR photons to maintain high efficiency [20]. The PCEs of ST-OSCs are intimately connected to the characteristics of transparent electrodes on both sides of the devices. By tuning the reflectivity of the top electrode, bifacial ST-OSCs may achieve higher PCE due to their ability to harness the incident light from both sides [21,22].

Recently, NIR-absorbing NFA and polymer donors demonstrated a new trend in ST-OSCs. Significant achievements in performance and transparency have been realized by utilizing both narrow-bandgap polymer donors and NFAs as photoabsorbent materials. Consequently, understanding the significance of these newly designed materials in enhancing the PCEs and AVTs of ST-OSCs has become imperative. Hence, in this review, we present the recent progress in the ST-OSC field in terms of materials engineering and various approaches to enhance performance. This review is concentrated only on the progress in solution-processed bulk heterojunction single-junction ST-OSCs. This review begins by introducing the fundamentals and essential parameters in the development of ST-OSCs. Thereafter, we will provide contemporary indacenodithiophene (IDT)- and indacenodithienothiophene (IDTT)-based fused ring electron acceptor (FREA) NIR-absorbing materials that exhibit promising performance in ST-OSCs. Subsequently, we will provide material design strategies and recent progress in fused cyclopentadithiophene (CPDT)- and benzodithiophene (BDT)-based FREAs. Then, we will outline the comprehensive progress of Y6 and its derivatives, NIR photoabsorbent materials, that show outstanding performance in ST-OSCs. Then, we will summarize other recently developed small molecules that have efficient NIR-absorbing properties for potential use in ST-OSCs. Next, a brief discussion about design strategies for polymer donors and multicomponent strategies to enhance the PCE and AVT of ST-OSCs are provided. Finally, we present the outlook on the challenges that must be addressed to commercialize this fascinating technology. 

## 2. Fundamentals of ST-OSCs

In this section, we provide the figure-of-merit parameters to represent the performance of ST-OSCs. For optimizing the ST-OSCs, key factors, like average visible transmittance (AVT), color-rendering index (CRI), and light utilization efficiency (LUE), should be considered together with PCE. 

### 2.1. Power Conversion Efficiency (PCE)

The PCE is a critical metric in solar cell technology that determines the effectiveness of converting incident sunlight into electrical energy. The PCE of ST-OSCs represents how much incident light is converted to electricity while allowing for a degree of transparency in the visible region. The PCE is calculated as the ratio of the electric power output (*P_out_*) of a solar cell at its maximum power point (V_max_ × J_max_) to the power input of incident light (*P_in_*). The fill factor (FF) is calculated as the ratio of the maximum power output to the product of open-circuit voltage (V_oc_) and short-circuit current density (J_sc_). According to the below equation, the PCE is proportional to the values of V_oc_, J_sc_, and fill factor (FF), which means that all three factors need to be maximized simultaneously to enhance the device’s performance. The measurement of PCE is commonly conducted under simulated standard one-sun test conditions (1000 W/m^−2^, AM 1.5 G) [23].

$$PCE\;\left(\eta \right)=\frac{P_{out}}{P_{in}}=\frac{V_{OC}{\;\times \;J}_{SC}\;\times \;FF}{P_{in}}$$

### 2.2. Average Visible Transmittance (AVT)

**Figure 2:**
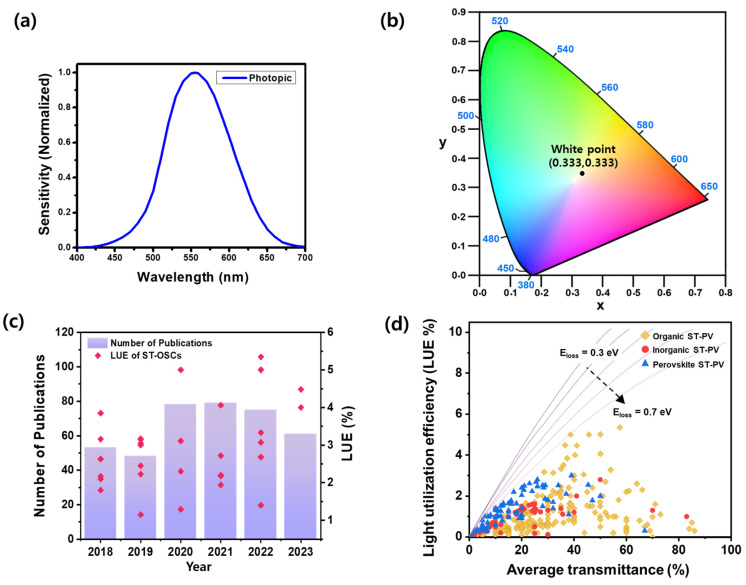
(**a**) Photopic spectral response of the human eye; (**b**) Commission Internationale de l’Eclairage (CIE) 1931 chromaticity diagram; (**c**) progress in the LUE and publication numbers of ST-OSCs since 2018. A number of publications were retrieved from the Web of Science (keyword: ST-OSCs), and the LUE values are shown in Table 1, Table 2 and Table 3. (**d**) Comparison of selected results of LUE and AVT for the organic, inorganic, and perovskite solar cells.

The second most important parameter of ST-OSCs is AVT. Unlike opaque solar cells, ST-OSCs must also allow a certain amount of light to pass through. Due to the sensitivity of the human eye, the transmittance of the device is only described in terms of the visible light between 370 and 740 nm [24]. The calculation of AVT is determined according to the following equation [25]

$$AVT=\frac{\int T\left(\lambda \right)V\left(\lambda \right)S\left(\lambda \right)d\lambda }{V\left(\lambda \right)S\left(\lambda \right)d\lambda }$$

where *T* represents the light transmittance, *λ* is the wavelength of light, *V* is the photopic response of the human eye, as shown in Figure 2a, and *S* shows the solar photon flux. It is imperative to systematically adjust the transmittance of ST-OSCs during fabrication to match the necessary transmittance for diverse applications. It is widely accepted that the minimum requirement for window applications for ST-OSCs in BIPVs is an AVT of 25% [26,27]. 

### 2.3. Color Perception

In semitransparent solar cell technology, color perception is another important aspect to determine its optical properties. The International Lighting Commission (CIE) 1931 chromaticity diagram shown in Figure 2b, specially designed for human color perception, can be used to determine the transparency and color properties of a measured spectrum [28]. Despite the fact that colorful ST-solar cells may be good for esthetical purposes, neutral color coordinates of ST-SCs close to “white point” (0.3333, 0.3333) or standard day light (D65) illuminants (0.3128, 0.3290) and the AM 1.5G light spectrum (0.3202, 0.3324) are typically chosen for window applications because the natural light environment will not be affected too much [20,22]. The color-rendering index (CRI), proposed by the Colsman group, is another crucial parameter for ST-OSC devices [29]. The CRI refers to the quality of a light source in terms of its ability to accurately reproduce the object’s color in transmitted light relative to natural light. CRI values generally range from 0 to 100, and a higher CRI (>85) value of ST-OSCs would closely resemble the system, accurately representing colors in their true form with superior color-rendering capacity [29,30]. 

### 2.4. Light Utilization Efficiency (LUE)

To provide the trade-off correlation between PCE and AVT, the comparison of technologies against theoretical limits is facilitated by the concept of light utilization efficiency (LUE = PCE × AVT), proposed by Lunt’s group [31]. The LUE value serves as a convenient key metric to compare the ST-OSC performance. In this context, the strategic design of organic photoactive materials with narrow-bandgap properties and wavelength selectivity, particularly in the NIR region, emerges as a key and pivotal factor in achieving efficient ST-OSCs. So far, between 5–15% PCE, 40–60% AVT, and 3–4% LUE have been demonstrated for most ST-OSCs across the literature [22,32]. Over the years, the number of publications and LUE of ST-OSCs have continued to increase, and state-of-the-art ST-OSC LUEs achieved ~5%, as shown in Figure 2c,d. 

### 2.5. Quantum Utilization Efficiency (QUE)

In OSCs, external quantum efficiency (EQE) is commonly used to characterize photon-to-electron conversion efficiency. However, photons within the visible-light range that pass through the ST-OSC device are beneficial to improving the AVT. Hence, for the ST-OSCs, the photon utilization efficiency is assessed using a determined factor, known as QUE. The calculation for QUE is defined as QUE = EQE + T, where *T* is the transmittance of ST-OSC. Owing to the intrinsic loss of photon and carrier (parasitic absorption, non-radiative recombination, interfacial reflection, etc.) in ST-OSCs, the QUE value must be lower than 90% in the whole spectral region [25,33].

### 2.6. Bifaciality Factor

It is possible to harvest photons from both the front and rear sides of ST-OSCs due to their high transmittance characteristics. However, there can be differences in PCE between top/bottom electrodes due to variations in the transmittance and reflectivity of the materials used for interfacial layers and top/bottom electrodes. Under the same irradiance, the bifaciality factor is expressed as the ratio of front PCE to rear PCE. In an ideal ST-OSC device, the bifaciality factor should be 1, signifying equal efficiency from both sides when illuminated with the same irradiance. 

## 3. Selection of Photoabsorbent Materials for ST-OSCs

The active layer materials that absorb light are core components of ST-OSCs and have a direct impact on the PCE and transparency. Generally, the photoactive layer of ST-OSC is made of organic donor and acceptor molecules in a bulk heterojunction structure. During the past two decades, fullerene-based acceptors have made significant progress and are widely used in OSCs due to their strong electron-withdrawing ability, isotropic behavior, and superior electron transport nature [34,35]. But, due to the limited energy level modification, high voltage loss, high cost, and inherent drawbacks of the absorption in the NIR region, fullerene-based acceptors are limited in terms of further developments in ST-OSCs [36,37]. Nevertheless, some conventional classic active material combinations, such as P3HT:PC_61_BM, PM6:PC_71_BM, and PTB7-Th:PC_71_BM, have been utilized in ST-OSCs [22,25,28]. Also, fullerene-based devices with some low-bandgap polymers showed a moderate PCE of ~5%, with an efficient AVT of 45–60%. For instance, a PBDTT-SeDPP polymer (bandgap 1.38 eV) with PC_61_BM acceptor achieved an ST-OSC PCE of 4.6% with an AVT of ~60%. Further, when combined with the PC_71_BM acceptor, the device showed a PCE of 5.5% due to enhanced absorption of the visible region by the PC_71_BM acceptor [38]. In both devices, nearly half of the NIR absorption in the 650–800 nm range was not utilized for energy conversion, indicating the necessity for strong absorption materials in the NIR region for efficient ST-OSCs. For ST-OSCs to achieve high performance, the ideal active layer should comprise a donor and acceptor, both showing a narrow bandgap with strong absorption in the NIR region while being transparent in the visible region [39,40]. In the below section, we will discuss the recent progress in narrow-bandgap NFA photoactive absorber materials in ST-OSCs.

### 3.1. Current Progress in Non-fullerene Acceptors 

At the beginning of OSC research, a wide-bandgap organic semiconductor material was typically used, and these materials can only use UV and visible light, so the ST-OSC performance based on these materials was found to be unsatisfactory. The Shockley–Queisser (SQ) limit for the PCE of a single-junction opaque OSC is 33.1%, while 20.6% for an ST-OSC with an AVT of 100% [41]. Small molecules emerge as competitively feasible to their polymer counterparts because of several advantages, such as less batch-to-batch variation, easier energy level control, defined molecular weight, and ascertainable structure–property relationships with optoelectronic properties [42,43]. The advent of ITIC- and Y6-based NFA materials in 2015 and 2019, respectively, resulted in rapid progress in fused ring electron acceptors (FREAs) with NIR absorption and offered a new opening to achieve a good balance between the PCE and transparency of ST-OSCs [44,45]. 

#### 3.1.1. Indacenodithiophene (IDT)- and Indacenodithienothiophene (IDTT)-Based Acceptors

FREAs have several advantages, such as tailoring of frontier energy levels, easy control of crystallinity, and low synthetic cost, over fullerene-based acceptors [46]. The rigid, high co-planar, and strong intermolecular interaction of FREAs plays a crucial role in reducing rotational disorder and reorganization energy and, thus, enables π-electron delocalization, superior charge mobility, and absorption towards the NIR region, with low energy loss (E_loss_) [47,48]. 

In 2015, the Zhan group developed a planar NFA, IDT-2BR, by using indacenodithiophene (IDT) as the central donor core, and benzothiadiazole (BT) and 3-ethylrhodanine as the π-bridge and acceptor group, respectively. The compound exhibited extensive absorption, relatively high electron mobility, and effective phase separation, resulting in a PCE of 5.12% when combined with P3HT [49]. Benefiting from this work, the group reported the very first ST-OSC devices by using the IDT-2BR NFA molecule with a P3HT polymer donor and showed a PCE of 3.22%. The low PCE of the semitransparent device is mainly due to the low Jsc, FF, and increased series resistance compared to the opaque device [50]. In 2016, Sarah et al. designed and reported an efficient NFA, IDT-BR, to improve the performance of OSCs. Due to the well-matched morphological and optoelectrical properties of IDT-BR with a P3HT donor, the group achieved a notable PCE of 6.4%, which was the highest PCE with P3HT:NFA at that time [51]. Later, Chen et al. reported an efficient OSC with PCE over 11% by using an O-IDT-BR (also known as IDT-BR) NFA. The PvBDTTAZ polymer showed excellent film morphology with IDT-BR and an efficient acceptor crystalline domain, thus yielding a Voc of 1.08 V and a low voltage loss of 0.55 V [52]. Further, the Lee group fabricated PTB7-Th:IDT-BR ST-OSC devices by using a promisingly highly conductive and transparent top electrode. The device showed a PCE of 6.32% with an AVT of 35.4% [53]. 

In 2015, the Zhan group reported a milestone small-molecule NFA, ITIC, with fused rigid indacenodithienothiophene (IDTT) as the central core and 1,1-dicyanomethylene-3-indanone (IC) as the end-capped acceptor. Strong absorption in the ≈600−800 nm range with an optical bandgap (E_g_) of 1.59 eV was found, with highest occupied molecular orbitals (HOMOs) and lowest unoccupied molecular orbitals (LUMOs) of −5.48 and −3.83 eV, respectively. The optimized device of the ITIC NFA with PTB7-Th polymer donor achieved an efficient PCE of 6.85, which was a new record at that time for a fullerene-free OSC using a small-molecular acceptor and, hence, assumed to be a proper candidate for ST-OSC applications [44]. Liu et al. reported a new polymer donor, reg-PThE, with an ITIC acceptor and demonstrated a PCE of 8.69% for an ST-OSC with an AVT of 24.2% [54]. The improved intermolecular ordering resulted in enhanced crystallinity and electron mobility of NFAs. The bulkier phenylhexyl side chains of the ITIC were replaced by a linear octyl chain to increase the crystallinity and reduce the bandgap in C8-ITIC compared to the ITIC. The delicate optical engineering and device optimization for the fabricated ST-OSTs showed a PCE of 9.8% with an AVT of 22% [55]. 

**Figure 3:**
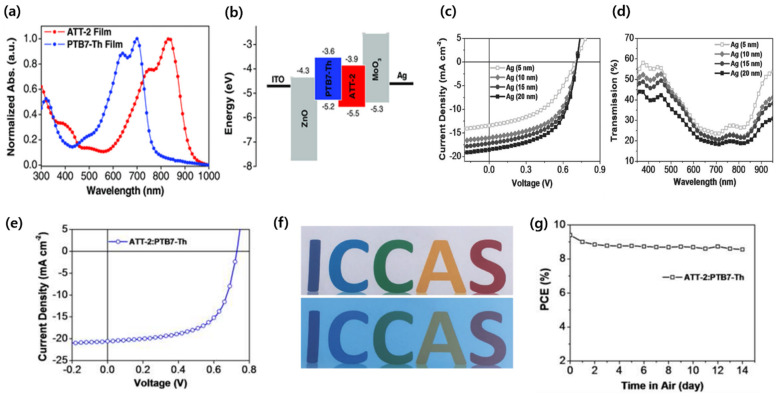
(**a**) Absorption spectra of PTB7-Th and ATT-2 in thin films; (**b**) energy level diagrams; (**c**) *J–V *curves; (**d**) transmission spectra of the devices; (**e**) *J–V* curve of the OSC device with active area of 0.1 cm^2^; (**f**) photographs of letters without and with the semitransparent device; and (**g**) the stability of OSC device efficiencies in ambient conditions. Reproduced with permission from [57]. Copyright 2017, John Wiley and Sons.

A highly effective design strategy for photoactive materials is to use and stabilize their quinoidal resonance structures and extend the π conjugation across the backbone. The ester group containing NFA molecules exhibits very strong absorption, simultaneously decreasing the frontier orbital energy level due to their strong electron-withdrawing ability. Zhu and co-workers developed an ester group containing a thieno[3,4-b]thiophene-based electron acceptor, ATT-1. The TbT (ester substituted) and 2-(1,1-dicyano-methylene) rhodamine units employing ATT-1 showed a strong absorption with λ_max_ of 736 nm (E_g_ of 1.54 eV) and simultaneously downshifting the HOMO and LUMO energy levels. The device based on PTB7-Th:ATT-1 delivered an outstanding PCE of 10.07%, which was the best-reported PCE at that time using PTB7-Th as the electron donor for single-junction OSCs [56]. Inspired by their previous work, the group reported another small-bandgap (1.32 eV) NFA material, ATT-2, replacing the end-group acceptor with stronger electron-withdrawing ability. The ST-OSC device showed a PCE of 7.7%, AVT of 37%, a high CRI of around 94, and a CIE color coordination (0.2805, 03076) (Figure 3a–g) [57]. These important findings suggest that the augmented ICT effect by employing stronger electron-withdrawing group led to a higher efficiency for ST-OSCs.

The π-spacer unit also plays a key role in tuning the optoelectronic properties, extending the absorption and molecular arrangement behavior. Oligothiophene-containing alkyl, alkoxy, or alkyl thio (R, OR, SR) units provide good solubility for solution processing; tuning the optoelectronic properties was successfully explored in NFAs for efficient OSCs [58,59]. For instance, Hou’s group synthesized a narrow-bandgap (1.34 eV) molecule, IEICO, by introducing an alkoxy thiophene unit as a π-spacer compared to its analogous molecule IEIC (1.57 eV). The reduction in the bandgap of IEICO is mainly due to the upshift in the HOMO energy level after using an electron-rich alkoxy group (vs. alkyl group) in the π-bridge [60,61]. 

Another useful approach for designing NIR materials is to employ the halogen atoms in electron-withdrawing acceptor units [62,63]. Fluorine (F), the most electronegative element (3.98 on the Pauling scale) in the periodic table, is only 20% larger in size than hydrogen. The comparatively small van der Wall radius of F (147 for F pm vs. 120 pm for H) provides a natural choice for the synthetic chemist to design and adjust the optoelectronic characteristics of materials and has been widely used in efficient OSCs [64]. The F atom provides an inductive (*σ*) electron-withdrawing effect without undesirable steric hindrance, significantly increasing the planarity and ordered intermolecular packing, in addition to lowering the frontier energy level. The non-covalent interactions, such as F···H, F···π, and F···S, exert a strong influence on the photophysical and crystalline properties of fluorinated molecules and their thin-film base device performance. Furthermore, the increased dipole moment of the C-F bond also extends the absorption range and reduces bandgaps in the NFAs by intensifying the ICT effect. Therefore, lots of research groups have designed and reported diverse new NIR-absorbing NFA materials to fabricate OSCs/ST-OSCs [65]. In addition to fluorination, chlorinated (Cl) end-group acceptors in NFA materials also emerge as a new star in OSCs. Chlorination proves to be more effective in modulating the optoelectrical characteristics of OSC materials than fluorination. In numerous cases, the energy level of chlorinated materials is lower, and their absorption spectrum is broader than that of fluorinated materials, which probably happens due to a larger dipole moment from the C-Cl bond than C-F (2.943 vs. 2.851). The increased overlap of π-electrons facilitated by Cl benefits intermolecular packing and crystalline properties, thus improving charge transport and performance. For example, in 2019, Cui et al. modified the well-known acceptor Y6 by replacing fluorine with chlorine (BTP-4Cl), which showed an extended absorption of 20 nm due to enhanced ICT effect lowering both the HOMO and LUMO energy levels (0.1 eV for LUMO and 0.03 eV for HOMO). Interestingly, this reduced the nonradiative voltage loss, and the corresponding devices yield an outstanding PCE of over 16%, which was the highest efficiency at that time for single-junction OSCs [66]. 

**Figure 4:**
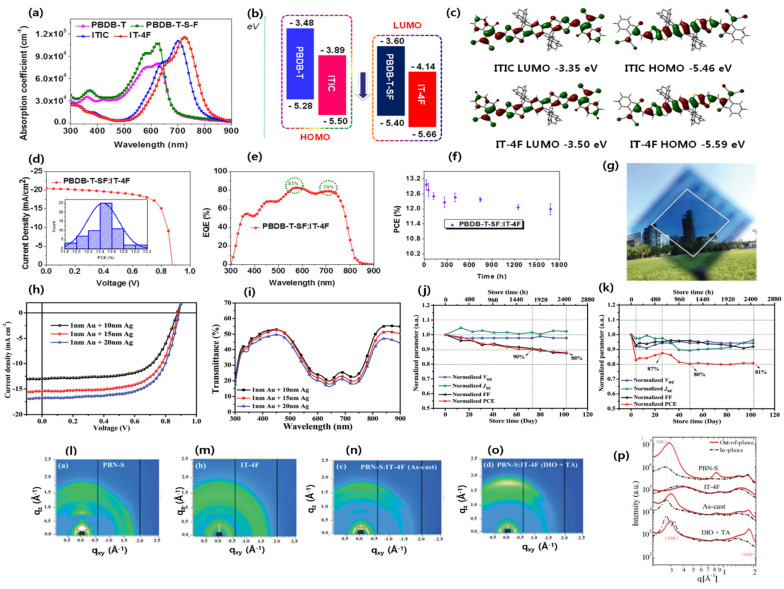
(**a**) Absorption spectra; (**b**) molecular energy levels; (**c**) DFT calculation of energy levels of donors and acceptors; (**d**) *J–V* curve and histogram (inset) of the PCE measurements for PBDB-T-SF:IT-4F-based OSCs; (**e**) EQE curve; and (**f**) storage stability of the PBDB-T-SF:IT-4F-based OSCs. Adapted with permission from [68]. Copyright 2017, American Chemical Society. (**g**) Photograph; (**h**) *J–V* curve and (**i**) transmittance spectra of PBN-S:IT-4F-based ST-OSCs. (**j**) Unencapsulated devices with a conventional configuration of glass/ITO/PEDOT:PSS/PBN-S:IT-4F/ZnO/Al stored in a nitrogen-filled glove-box; (**k**) unencapsulated devices with an inverted configuration of glass/ITO/ZnO/PBN-S:IT-4F/MoO_3_/Al stored in air; 2D-GIWAXS patterns (**i**–**o**); and (**p**) in-plane and out-of-plane of the neat film and the PBN-S:IT-4F. Reproduced with permission [69]. Copyright 2019, Royal Society of Chemistry.

Li and co-workers designed and synthesized a new NIR-absorbing low-bandgap (LBG) NFA ID-4Cl by using a strong electron-withdrawing IC–2Cl acceptor group. The ID-4Cl shows main absorption in the 600–900 nm region due to a strong ICT effect between the IDT core and 2-(5,6-dichloro-3-oxo-2,3-dihydro-1H-inden-1-ylidene)malononitrile (IC-2Cl). The fabricated ST-OSC device with a PM6 polymer yields a PCE of 6.99% with an AVT of 43.7% [67]. Zhao et al. designed and synthesized a new NFA, IT-4F (also known as ITIC-4F), by introducing four fluorine atoms on ITIC. The material showed a downshifted HOMO/LUMO energy level (−5.66/−4.14 eV) compared to their nonfluorinated ITIC counterpart (−5.50/−3.89 eV) (Figure 4a–c). The fabricated OSC device reached an outstanding PCE of 13.1% due to its higher absorption coefficient, strong intermolecular interaction, improved crystallinity, and, hence, facilitated charge transport than nonfluorinated molecule Figure 4d–f [68]. Wu et al. synthesized a new fluorine-containing dialkylthio-substituted polymer, PBN-S, blended with ITIC-4F. Further, 2D grazing incidence wide-angle X-ray scattering (2D-GIWAXS) measurements showed stronger π-π stacking after optimization (1% DIO and thermal annealing) than the as-cast blend, resulting in a higher charge mobility and fill factor. The ST-OSC device demonstrated a PCE of 9.83%, with an AVT of 32% and a notable device stability, retaining around 80% PCE after storage in a nitrogen glove-box for 100 days (Figure 4g–p) [69].

In 2017, Li et al. reported a series of narrow-bandgap NFAs by using double-bond π-bridges into ITIC (ITVIC) with monofluorine (ITVfIC) or bifluorine (ITVffIC) substituents on the ITIC end group. Due to extending the π-conjugation and increasing additional ICT effects by fluorine atoms, all three molecules reduced the bandgap from 1.40 to 1.35 eV compared to ITIC (1.59 eV). The ITVffIC-based device showed an efficient PCE of 10.54%, with a remarkably low energy loss of 0.54 V, demonstrating promising application in ST-OSCs [70]. Further, the group utilized ITVfIC in ST-OSC, which shows an absorption peak at 772 nm with a corresponding bandgap of 1.37 eV. The fabricated ST-OSC using a PTB7-Th donor showed a PCE of 8.21%, with an AVT of 26.4%. In addition, the device’s PCE value retains 91% after 2 h at 200 °C, indicating good thermal stability [71]. Zhang et al. synthesized a series of symmetric/asymmetric NFAs, 6-IFIC, 6-IF2F, and 6-IF4F, containing fluorinated thieno[3,4-b]thiophene. The π-bridge unit extends the absorption and planarizes the molecular geometry, while the asymmetric structure provides a larger dipole moment, thus enhancing the intermolecular interaction and resulting in an efficient ST-OSC PCE of 7.87% with an AVT of 28.04% based on PTB7-Th:6-IF2F [72].

Yao et al. introduced fluorine atoms into the IC end-group acceptor of IEICO and designed IEICO-4F. The device based on binary blended PBDTTT-EFT:IEICO-4F showed a PCE of 10.0% with a high Jsc of 22.8 mA cm^−2^ because of the synergistic enhancement in NIR absorption, strengthening the ICT effect [73]. The main absorption peak of neat PTB7-Th is located around 700 nm, which leads to the obstacle of high transparency. In this way, the lower content of the polymer donor could yield high AVT. Therefore, Zhang’s group decreased the photon absorption in the visible region and simultaneously improved the photon harvesting in the NIR region by changing the PTB-7Th:IEICO-4F ratios from 1.4:1.5 to 0.8:1.5 *wt*/*wt*, resulting in an increased AVT of 27.1% with an impressive PCE of 9.06% for ST-OSC [74]. Further, Cui et al. replaced the fluorine atoms of IEICO-4F with chlorine atoms and constructed ultralow-bandgap (1.23 eV) NFA, IEICO-4Cl. The enhanced ICT effect due to the high dipole moment of Cl atoms compared to F further red-shifted the absorption, making is useful for ST-OSCs. Thus, the device based on the three different polymer donors and IEICO-4Cl (J52:IEICO-4Cl, PBDB-T:IEICO-4Cl, and PTB7-Th:IEICO-4Cl) yields variable color solar cells from purple and blue to cyan. Among them, the optimized device for PTB7-Th:IEICO-4Cl produced an efficient PCE of 8.38% with an AVT of 25.7% [75].

**Figure 5:**
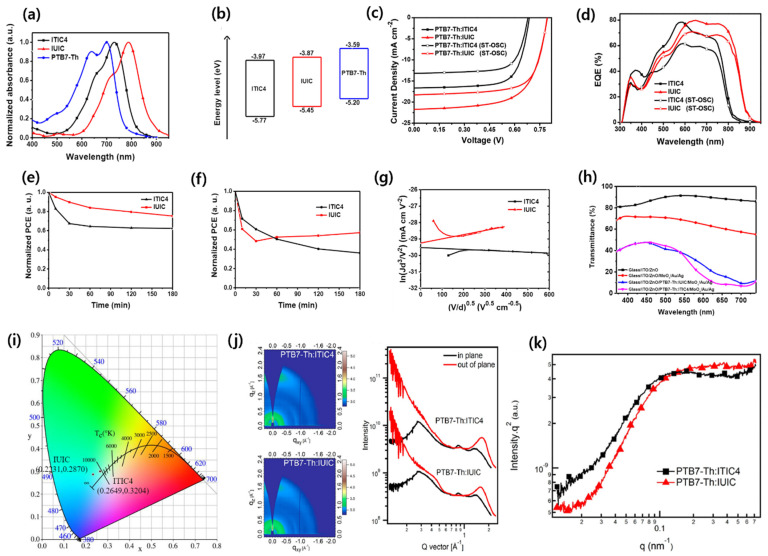
(**a**) Thin-film absorption spectra; (**b**) energy levels of PTB7-Th, IUIC, and ITIC4; (**c**) *J–V* curves; (**d**) EQE spectra of optimized OSC and ST-OSC as-cast devices based on PTB7-Th:ITIC4 and PTB7-Th:IUIC; (**e**) stability of the OSC devices based on PTB7-Th:IUIC and PTB7-Th:ITIC4 under AM 1.5G illumination at 100 mW cm^−2^ and (**f**) under heating at 100 °C. (**g**) *J–V* characteristics in dark for electron-only devices based on IUIC and ITIC4; (**h**) visible transmission spectra of with PTB7-Th:IUIC and PTB7-Th:ITIC4 with and without active layer, and (**i**) CIE 1931 color coordinate diagram of IUIC and ITIC4. (**j**) 2D GIWAXS patterns and scattering profiles of in-plane and out-of-plane for PTB7-Th:IUIC and PTB7-Th:ITIC4/IT-4F blended films; (**k**) R-SoXS profiles in log scale for PTB7-Th:IUIC and PTB7-Th:ITIC4 blended films. Reproduced with permission [80]. Copyright 2018, American Chemical Society.

Lee et al. designed and synthesized a series of NIR absorber NFA materials, ITIC-2F, IOTIC-2F, and ITOTIC-2F. The OSC device based on the PTB7-Th:IOTIC-2F blend achieved an outstanding PCE of 12.1% due to nanoscale phase separation and good crystallinity features of NFA molecules. The achieved performance includes a high Jsc of 22 mA cm^−2^, Voc of 0.82 V, and a notable Eloss of around 0.49 V, despite a very small HOMO offset (∆EH) of only 0.02 eV [76]. In addition, Zou et al. synthesized a fused-ring narrow-bandgap NFA, IE4F-S (also known as IEICS-4F), and the device yielded an efficient PCE of 12.2% with 0.996 V and a notably low Eloss of 0.47 eV when blended with a PTQ10 polymer donor despite the negligible ∆EH [77]. Further, the same group also synthesized and reported a new low-bandgap acceptor, TPT10, and achieved an outstanding PCE of 16.32% with a PTQ11 polymer donor, with an impressive zero ∆EH [78]. These works suggested that small ∆EH is not an essential limitation in realizing the high efficiency of NIR NFA-based solar cells. Yan and co-workers also replaced the alkoxy (OR) group with the alkyl thio (SR) group of IEICO-4F and found that the introduction of alkylthio in IEICS-4F increased the crystallinity and, thus, improved the electron mobility and resulted in a PCE of 7.5% with an AVT of 35% for the ST-OSC binary blend with a PTB7-Th polymer donor [79]. Jia et al. designed and synthesized one of the largest fused cores containing an NFA molecule, namely IUIC. As expected, the fused undecacyclic (11-member rings) exhibited a strong ICT effect between the large π-conjugation core and 2-(5,6-difluoro-3-oxo-2,3-dihydro-1H-inden-1-ylidene)malononitrile (IC-2F), leading to a higher absorption coefficient of 3 × 10^5^ cm^−1^ and upshifted LUMO energy level of –3.87 eV than ITIC4 (also known as IT-4F and ITIC-4F) (Figure 5a–k). The resonant soft X-ray scattering (R-SoXS) indicated that relative domain purity of the PTB7-Th:IUIC blend, being higher than PTB7-Th:ITIC4 and so minimizing the possibility of bimolecular recombination and facilitating the charge transfer. The ST-OSC device based on the PTB7-Th:IUIC demonstrated an efficient PCE of 10.2%, AVT of 31%, and color coordinate of (0.2231, 0.2870) due to extending absorption in the NIR region and high electron mobility (μe = 1.1 × 10^−3^ cm^2^ V^−1^ s^−1^). These findings indicate that incorporating larger fused ring core NFA could be an effective strategy in extending the absorption in the NIR region as well as for a performance improvement [80].

**Figure 6:**
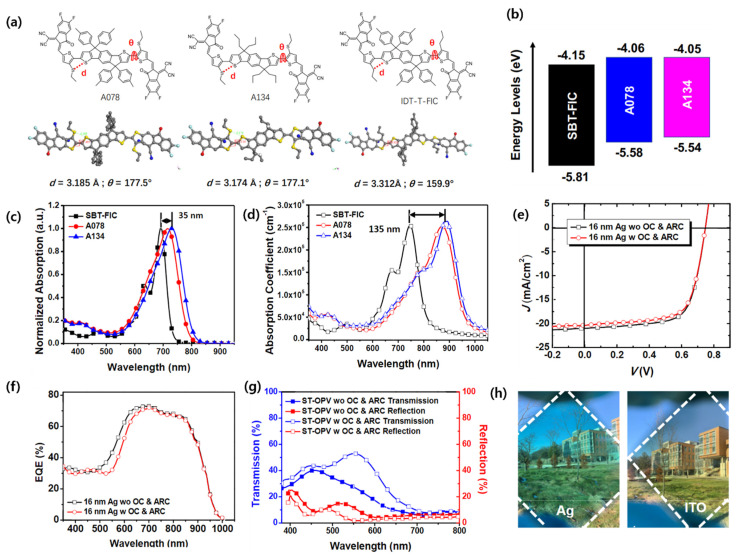
(**a**) Molecular geometry calculated by DFT and torsional angles (*θ*) between the flanking thiophene and the IDT core. The *d* is the S···S distance (A078 and A134) or C···S distance of (IDT-T-FIC); (**b**) energy level diagram of SBT-FIC, A078, and A134; (**c**) UV-vis absorption spectra of SBT-FIC, A078, and A134 in toluene solution and (**d**) in thin film. (**e**) *J–V* and (**f**) EQE curves; (**g**) optical transmission and reflection of the optimized ST-OSC with and without outcoupling (OC) and antireflection coating (ARC), and (**h**) outdoor images of ultrathin semitransparent device. Reproduced from [82].

**Figure 7:**
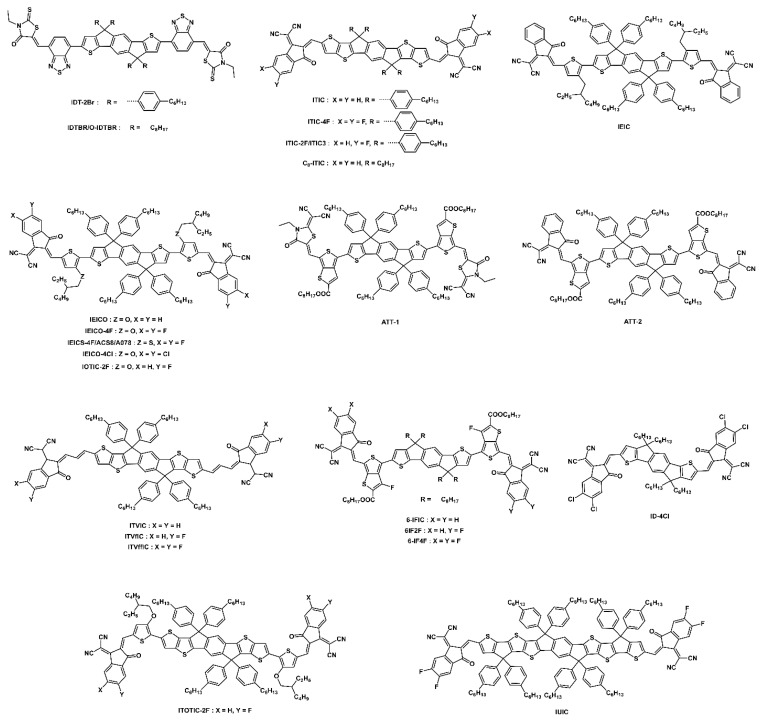
Molecular structures of IDT- and IDTT-based NFA.

Zhang’s group designed and synthesized an ultranarrow-bandgap (1.3 eV) NFA, ACS8, by replacing the alkoxy side to alkylthio chain on the π bridge of IEICO-4F. The ACS8 NFA demonstrated good electron mobility of 2.65 × 10^−4^ cm^2^ V^−1^ s^−1^ (1.14 × 10^−4^ cm^2^ V^−1^ s^−1^ for IEICO-4F), owing to its well-ordered molecular orientation and compact π-π stacking. The ST-OSC device based on the PTB7-Th:ACS8 (1:2, *w*/*w*) with Ag thickness of 20 nm achieved an outstanding PCE of 11.1%, with high Jsc of 22.5 mA cm^−2^, AVT of 28.6%, a CRI of 84, and CIE color coordinates of 0.2621, 0.2976 [81]. Similarly, Li et al. also designed and reported the ACS8 NFA independently (named A078). The group further compared the absorption of A078 with some modified counterparts (SBT-FIC and A134). Interestingly, A078 exhibited a notable red shift (135 nm) compared to the more fused ring member SBT-FIC, clearly indicating that A078 has a more rigid and planarized structure due to S···S interaction and compact molecular packing behavior than the SBT-FIC molecule. The ST-OSC device with a PCE-10:A078 blend achieved a high PCE of 11%, AVT of 25%, LUE of 2.8%, and CIE color coordinates of 0.27, 0.34. Moreover, the authors further optimized the device by fabricating four-layered outcoupling coating on the Ag anode and, subsequently, two-layer antireflective coating (ARC) on a glass substrate, demonstrating a PCE 10.8%, AVT of 45.7%, CIE of 0.33, 0.39, and a record high LUE of 5% (Figure 6a–h). [82]. The chemical structures of some promising IDT and IDTT-based electron acceptor materials are shown in Figure 7, and their optoelectrical properties and solar cell performance are summarized in Table 1. 

**Table 1:** Optoelectronic properties and photovoltaic parameters of IDT- and IDTT-based materials.

Device Structure ^a^	NFA	HOMO eV	LUMO eV	E_g_^opt^ eV	Voc V	Jsc mAcm^2^	FF %	PCE % (ST-OSC)	PCE % (OSC)	AVT %	LUE %	CRI	CIE 1931 (x, y)	Ref.
Glass/PES/PH1000/PEIE/P3HT:IDT-2BR/PH1000-T	IDT-2BR	−5.52	−3.69	1.68	0.84	6.23	62	3.22	4.20	50	1.61	−	−	[50]
ITO/ZnO/P3HT:IDT-BR/MoO_3_/Ag	IDTBR	−5.51	−3.88	1.63	0.72	13.9	60	−	6.30	−	−	−	−	[51]
glass/ITO/ZnO/PvBDTTAZ:O-IDTBR/V_2_O_5_/Al	O-IDTBR	−5.51	−3.88	1.63	1.08	16.26	63	−	11.6	−	−	−	−	[52]
ITO/ZnO NP/PTB7-Th:IDTBR/PIL	IDTBR	−	−	−	0.98	11.35	57	6.32	8.50	35.4	2.23	−	−	[53]
ITO/PEDOT:PSS/PTB7-TH:ITIC/PDIN/Al	ITIC	−5.48	−3.83	1.59	0.81	14.21	59	−	6.8	−	−	−	−	[44]
ITO/ZnO/reg-PThE:ITIC/MoO_3_/Ag	ITIC	−	−	−	0.91	14.28	66	8.69	−	24.2	2.1	−	−	[54]
ITO/ZnO/PFBFB-T:C8ITIC/ MoO_3_/Ag/MoO_3_	C8-ITIC	−5.63	−3.91	1.72 ^b^	0.92	17.34	70	9.8	−	22	2.15	−	−	[55]
ITO/PEDOT:PSS/PTB7-Th:ATT-1/PFN/Al	ATT-1	−5.50	−3.63	1.54	0.87	16.48	70	−	10.07	−	−	−	−	[56]
ITO/ZnO/PTB7-Th:ATT-2/MoO_3_/Ag	ATT-2	−5.50	−3.90	1.32	0.71	18.53	59	7.74	9.58	37	2.86	94.1	0.281, 0.307	[57]
ITO/PEDOT:PSS/PTB7 Th:IEIC/PDIN/Al	IEIC	−5.42	−3.82	1.57	0.97	13.55	48	−	6.31	−	−	−	−	[60]
ITO/PEDOT:PSS/PBDTTT-E-T:IEICO/PFN-Br/Al	IEICO	−5.32	−3.95	1.34	0.82	17.7	58	−	8.40	−	−	−	−	[61]
ITO/PEDOT:PSS/PM6:ID-4Cl/PDINO/Au	ID-4Cl	−5.81	−4.01	1.51	0.75	13.77	68	6.99	10.25	43.7	3.05	−	−	[67]
ITO/ZnO/PBDB-T-SF:IT-4F/MoO_3_/Al	IT-4F	−5.66 ^c^	−4.14 ^c^	1.52 ^c^	0.88	20.88	71	−	13.10	−	−	−	−	[68]
ITO/PEDOT:PSS/PBN-S:IT-4F/ZnO/Au	IT-4F	−	−	−	0.88	16.78	66	9.83	−	32	3.15	92	0.205, 0.231	[69]
ITO/PEDOT:PSS/J71:ITVIC/PDINO/Al	ITVIC	−5.46	−3.97	1.40	0.89	14.47	58	−	7.34	−	−	−	−	[70]
ITO/PEDOT:PSS/J71:ITVfIC/PDINO/Al	ITVfIC	−5.56	−4.01	1.37	0.84	19.73	59	−	9.72	−	−	−	−	[70]
ITO/PEDOT:PSS/J71:ITVffIC/PDINO/Al	ITVffIC	−5.58	−4.04	1.35	0.81	20.60	63	−	10.54	−	−	−	−	[70]
ITO/PEDOT:PSS/PTB7-Th:ITVfIC/PDINO/Ag	ITVfIC	−5.56	−4.01	1.37	0.74	17.54	63	8.21	−	26.40	2.17	−	0.29, 0.36	[71]
ITO/PEDOT:PSS/PTB7-Th: 6-IFIC/PDINO/Ag	6-IFIC	−5.34	−4.02	1.27	0.69	17.28	56	6.94	9.74	27.99	1.94	59	0.262, 0.318	[72]
ITO/PEDOT:PSS/PTB7-Th: 6-IF2F/PDINO/Ag	6-IF2F	−5.38	−4.07	1.25	0.64	20.05	59	7.87	11.20	28.04	2.20	60	0.263, 0.320	[72]
ITO/PEDOT:PSS/PTB7-Th: 6-IF4F/PDINO/Ag	6-IF4f	−5.42	−4.14	1.22	0.58	21.7	59	7.46	10.46	29.23	2.18	62	0.264, 0.312	[72]
ITO/PEDOT:PSS/PBDTTT-EFT:IEICO-4F/PFN-Br/Al	IEICO-4F	−5.44	−4.19	1.24	0.74	22.80	59	−	10.00	−	−	−	−	[73]
ITO/PEDOT:PSS/PTB7-Th:IEICO-4F/PDIN/Al	IEICO-4F	−	−	−	0.71	18.81	68	9.06	11.55	27.10	2.45	−	0.269, 0.292	[74]
ITO/PEDOT:PSS/J52:IEICO-4Cl/PFN-Br/Au	IEICO-4Cl	−5.56	−4.23	1.23	0.67	17.20	55	6.37	10.10	35.1	2.24	−	−	[75]
ITO/PEDOT:PSS/PBDB-T:IEICO-4Cl/PFN-Br/Au	IEICO-4Cl	−	−	−	0.72	15.40	56	6.24	9.67	35.7	2.23	−	−	[75]
ITO/PEDOT:PSS/PTB7-Th:IEICO-4Cl/PFN-Br/Au	IEICO-4Cl	−	−	−	0.73	19.6	59	8.38	10.30	25.6	2.15	−	−	[75]
ITO/ZnO/PTB7-Th:ITIC-2F/MoO_x_/Ag	ITIC-2F	−5.55	−4.15	1.56	0.75	16.2	70	−	8.7	−	−	−	−	[76]
ITO/ZnO/PTB7-Th:IOTIC-2F/MoO_x_/Ag	IOTIC-2F	−5.34	−4.06	1.31	0.82	21.9	65	−	12.1	−	−	−	−	[76]
ITO/ZnO/PTB7-Th:IOTIC-2F/MoO_x_/Ag	ITOTIC-2F	−5.22	−4.11	1.32	0.79	7.0	61	−	3.7	−	−	−	−	[76]
ITO/PEDOT:PSS/PTQ10:IE4F-S/PDINO/Al	IE4F-S	−5.54	−3.89	1.65 ^b^	0.99	19.67	62	−	12.2	−	−	−	−	[77]
ITO/ZnO/PTB7-Th:IEICS-4F/MoO_3_/Au	IEICS-4F	−5.43	−4.08	1.35	0.73	16.80	61	7.5	10.3	35	2.63	−	−	[79]
ITO/ZnO/PTB7-Th:IUIC/MoOx/Au/Ag	IUIC	−5.45	−3.87	1.41	0.79	18.31	70	10.2	11.2	31	3.16	75	0.233, 0.287	[80]
ITO/ZnO/PFN/PTB7-Th:ACS8/MoO_3_/Au/Ag	ACS8	−5.54	−4.05	1.30	0.74	22.5	67	11.1	13.2	28.6	3.17	84	0.262, 0.297	[81]
ITO/ZnO/PCE-10:SBT-FIC/MoO_3_/Ag	SBT-FIC	−5.81	−4.15	1.66 ^b^	0.70	18.1	62	−	7.9	−	−	−	−	[82]
ITO/ZnO/PCE-10:A134/MoO_3_/Ag	A134	−5.54	−4.05	1.49 ^b^	0.75	16.7	61	−	7.6	−	−	−	−	[82]
MgF_2_/ITO glass/ZnO/PCE-10:A078/MoO_3_/ITO/MgF_2_/MoO_3_/MgF_2_/Mo_3_	A078	−5.58	−4.06	1.52 ^b^	0.75	20.9	70	10.8	13.0	45.7	5.0	−	0.33, 0.39	[82]

^a^ Active layer was highlighted in blue color and photovoltaic parameters (Voc, Jsc, and FF) as well as the device structure were shown for ST-OSC when present both opaque and semitransparent device efficiency. ^b^ Calculated from CV measurements. ^c^ Obtained from the ultraviolet photoelectron spectroscopy (UPS) results.

#### 3.1.2. Fused Cyclopentadithiophene (CPDT)- and Benzodithiohene (BDT)-Based Acceptors

A simple and efficient design strategy for enhancing the intramolecular charge transfer (ICT) effect in narrow-bandgap NFAs is to increase the donating capacity of the central donor. A fused cyclopentadithiophene, IHIC (or 4TIC) NFA, was designed and synthesized by Zhang’s group and demonstrated an efficient ST-OSC efficiency of 9.77%, with an AVT of 36% with a PTB7-Th donor [83]. The π-system of the two fused cyclopentadithiophene facilitates more effective π-electron delocalization compared to the IDT core, due to a planar core and S···S interaction in the IHIC, which lower the resonance stabilization energy and rotational disorder, contributing to the expansion of the photoresponse range towards the NIR region. Further, by using two more thienyl groups in the IHIC core, Jen et al. extended the π-conjugation system and formed a narrower bandgap NFA, 6TIC, than IHIC. The 6TIC-based device with a PTB7-Th donor exhibited a PCE of 7.62% with an AVT of 23.3% [84]. It is noteworthy that highly efficient hole transfers occur from 6TIC to PTB7-Th, with minimal driving energies of approximately 0.01 eV. The voltage loss is remarkably low at only 0.55 eV. As demonstrated in this molecular design strategy, it is possible to reduce energy loss while maintaining a high Voc. An effective strategy for lowering NFA’s bandgap is to insert electron-rich oxygen atoms into its ladder-type donor backbone. By using a 7-heterocyclic-fused benzodithiophene (BDT) unit containing an alkoxy side chain, Lioa’s group developed a low-bandgap NFA, BT-IC (1.43 eV). The electron-rich alkoxy group in the side chains of the central donor core provides an efficient way to facilitate solution processing, extending the absorption profile. The OSCs fabricated with medium-bandgap polymers such as J61 and J71 showed efficient PCEs of 9.6% and 10.5% [85]. Interestingly, both device systems produced an effective charge even the HOMO offset (∆EH) of the blend was found to be low as 0.1 eV. 

**Figure 8:**
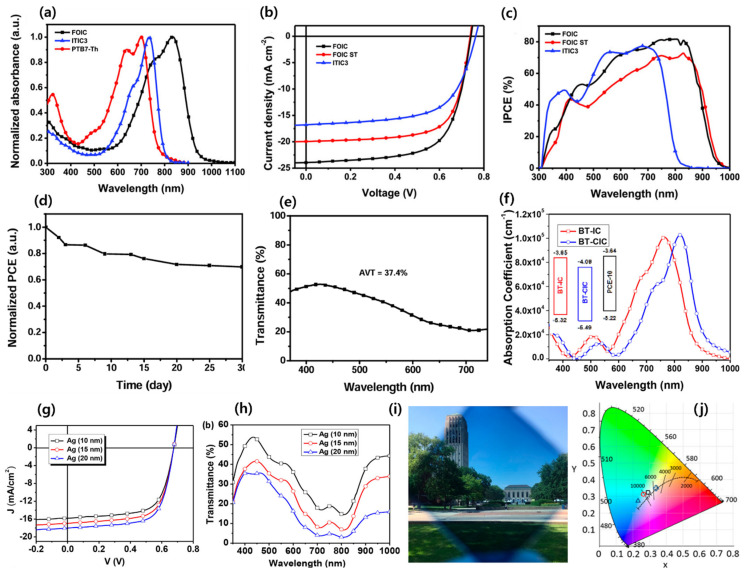
(**a**) UV-vis. absorption spectra of FOIC, ITIC3, and PTB7-Th in thin film; (**b**) *J–V* and (**c**) IPCE spectra of the best as-cast OSCs and ST-OSCs; (**d**) air stability of as-cast OSCs without encapsulation based on PTB7-Th: FOIC (1:1.5, *w*/*w*), and (**e**) visible transmission spectrum of the best as-cast ST-OSC. Reproduced with permission [86]. Copyright 2018, John Wiley and Sons. (**f**) UV–vis absorption spectra of BT-IC and BT-CIC thin films and energy level diagram of BT-IC, BT-CIC, and PCE-10 (inset); (**g**) *J–V* and (**h**) transmission spectra of ST-OSCs device based on PCE-10:BT-CIC (1:1.5, *w*/*w*) with different Ag thicknesses; (**i**) outdoor photograph of ST-OSC using 10 nm Ag thickness, and (**j**) CIE 1931 color coordinates of the transmission spectra of devices with different Ag thicknesses using a AM1.5G solar simulated input spectrum (denoted by ‘+’), 10 nm thick Ag (‘□’ square), 15 nm Ag (‘○’ circle), and 20 nm Ag (‘△’ triangle). Reproduced with permission [88]. Copyright 2017, American Chemical Society.

Zhang’s group modified the structure of 6TIC by introducing a fluorine atom at the 1,1-dicyanomethylene-3-indanone (IC) acceptor named FOIC. The FOIC showed stronger NIR absorption, resulting in a narrower bandgap of 1.32 eV compared to 6TIC (1.37 eV). Also, the FOIC showed a red shift in the absorption, reduced the bandgap and high molar extinction coefficient, and enhanced electron mobility compared to its IDTT core counterpart, ITIC3. The ST-OSC device performance for FOIC with a PTB7-Th donor demonstrated an efficiency of 10.3% and AVT of 37.4% (Figure 8a–e) [86]. Later, the same group reported a series of NFA molecules with the same strong electron-withdrawing IC-2F acceptor groups but fused 6, 8, and 10 ring central donor cores named F6IC, F8IC, and F10IC, respectively. All three NFAs showed strong absorption in the 600–1000 nm range with a high extinction coefficient and high electron mobility. Interestingly, the F8IC and F10IC molecules demonstrate very similar absorption in thin films, which signifies an expansion in the π-conjugation core that barely contributed to increasing the absorption extension. The OSC device based on the TB7-Th:F8IC revealed highest efficiency, 10.9%, with a high Jsc of 25.12 mA cm^−2^ due to its high mobility and superior π-π stacking among three NFAs. These findings suggest that, through the potential rational design of appropriate π-conjugated cores for NIR absorption, highly efficient ST-OSCs could be achieved [87].

On the basis of the BT-IC molecule, Forrest’s group reported BT-CIC NFA by substituting high-electron-affinity chlorine atoms in the IC group. The two adjacent Cl atoms on the acceptor considerably decreased both the HOMO and LUMO energy levels, with a stronger ICT effect, which extended the absorption in the NIR region and corresponded to a bandgap of 1.33 eV (for BT-IC 1.43 eV). The ST-OSC device with PTB7-Th revealed a PCE of 8.2% with an AVT of 26% due to improved stacking and ordered molecular aggregates through the Cl···Cl and Cl···S intermolecular interactions. In addition, when the Ag thickness of cathodes was controlled to 10 nm, a PCE of 7.1% with 43% of AVT, a CRI of 91, and color coordinates of 0.29, 0.32, nearly standard daylight conditions were observed, indicating that the light passing through these ST-OSCs closely resembles natural daylight in color (Figure 8f–j) [88]. 

#### 3.1.3. Y6-Based Non-Fullerene Acceptors

Recently, ladder-type NFAs with a A-DA′D-A-type structures have received increasing attention. As a result of the planar structure of the DAD core, electrons are delocalized via fused bridges within the core. In 2017, Feng et al. reported the first A-DA′D-A-type NFA, BZIC. By employing ladder-type fused cores through the insertion of an internal benzotriazole acceptor, BZIC achieved a remarkable red shift of 79 nm compared to ITIC due to increased planarization and intramolecular electronic interaction. Additionally, the BZIC film showed a higher absorption coefficient of 1.57 × 10^5^ cm^−1^ compared to ITIC (1.19 × 10^5^ cm^−1^) and delivered a solar cell PCE of 6.3% when used with the HFQx-T polymer. [89]. Similarly, by extending the fused core for Y1 and Y2 (modifying end-group acceptor also), the authors achieved PCEs of over 13%. Also, the devices based on Y1 and Y2 acceptors showed extremely low voltage loss values of 0.07 and 0.05 eV, respectively [90]. Later, based on the BZIC molecular design strategy, in 2019, Zou’s group designed and reported a well-known champion NFA molecule, Y6. By increasing the fused core and replacing the benzotriazole with electron-deficient benzothiadiazole (BT) of BZIC, the group successfully tuned the absorption and electron affinity of Y6. Consequently, the device based on a PM6:Y6 blend demonstrated a record-breaking efficiency of 15.7% and opened a new door in the molecular design of A-DA′D-A-type NFAs for high-performance OSCs [45]. 

**Figure 9:**
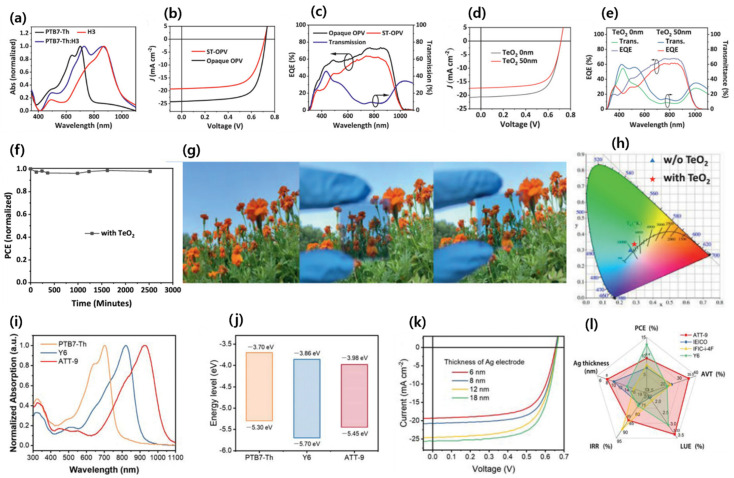
(**a**) Absorption spectra of PTB7-Th, H3, PTB7-Th:H3; (**b**) *J–V* spectra of PTB7-Th:H3; (**c**) EQE and transmission characteristic of PTB7-Th:H3; (**d**) *J–V* curves of ST-OSC without and with 50 nm TeO_2_; (**e**) EQE spectra and transmittance of ST-OPV without and with 50 nm TeO_2_; (**f**) stability of ST-OSC device with TeO_2_ (stored in glove box); (**g**) outdoor images of without ST-OSC device (left), ST-OSC with 0 nm TeO_2_ (center) and ST-OSC with 50 nm TeO_2_ (right); (**h**) The CIE 1931 color space of devices without or with 50 nm top TeO2. Reproduced with permission [94]. Copyright 2021, John Wiley and Sons. (**i**) Absorption spectra and (**j**) energy level diagram of PTB7-Th, Y6, and ATT-9; (**k**) *J–V* curves of PTB7-Th:ATT-9-based ST-OSC with different Ag thicknesses; (**l**) parameter comparison of ATT-9-based ST-OSC with IEICO, IFIC-i-4F, and Y6-based ST-OSCs. Reproduced with permission [95]. Copyright 2022, John Wiley and Sons.

Luo et al. designed and synthesized a low-bandgap (1.30 eV) NFA, Y14, by using a benzotriazole core and obtained an ST-OSC PCE of 12.67%, with an AVT of 23.69%. The inclusion of alkyl chains in the core donor improved the solubility and prevented the excessive self-aggregation of Y14 [91]. Zhang’s group reported ST-OSCs based on the PM6:Y6 and presumed that lowering the concentration of visible-absorbing PM6 could increase the AVT while maintaining the PCE. An efficient PCE of 7.46%, AVT of 36.4%, and LUE of 2.72% were achieved by using only 20 wt% polymer donors. This work suggests that combining a low amount of polymer donors with NIR NFA could achieve high PCE and AVT simultaneously [92]. Hu et al. modified the thickness of the Ag electrode and obtained an impressive PCE of 12.37% with an AVT of 18.6% for a PM6:Y6 ST-OSC device [93]. Li et al. reported a high-performance ST-OSC based on H3 NFA. An ultra-smooth Ag layer with a small granule size was created via polyethylenimine (PEI) wetting to improve the absorbing selectivity of the device (ITO/PEDOT:PSS/PTB7-Th:H3/ZnO/PEI/Ag/TeO_2_). Further, an optical interference layer, TeO_2_, was deposited over the ultrathin Ag layer with the guidance of optical simulations, and this resulted in a dramatic improvement in the absorbance selectivity by increasing the transmission in the visible region without significantly reducing absorption in the invisible region. As a result of the combined implementation of these strategies, they reported a high PCE of 8.38%, AVT of 50.09%, and LUE of 4.06%, with a good CRI of 76.85 and color coordinates of 0.291, 0.339 (Figure 9a–h) [94].

In ST-OSCs, the lack of balance between selective absorption and energy conversion of active layers results in an inferior LUE. Theoretically, it is possible to achieve a LUE of 20% by using both ideal energy conversion and selective absorption of the active layer [31]. Until now, the best LUE obtained around 5% for single-junction ST-OSCs. To make further improvements to ST-OSCs, it is necessary to find a better balance between energy conversion and selective absorption. Zhu and co-workers synthesized a novel NFA, ATT-9, and developed an external quantum efficiency (EQE) model to explore the maximum potential of molecular design on ST-OSC performance. The ATT-9 molecule contained a quinoidal resonance thieno[3,4-b]thiophene moiety with an electron-withdrawing fluorine atom, simultaneously narrowing the bandgap (1.15 eV) and constraining the HOMO upshift. The ST-OSC device with a PTB7-Th donor showed an excellent PCE of 9.37%, AVT of 35%, and LUE of 3.33% (Figure 9i–l) [95].

#### 3.1.4. Other Novel Narrow-Bandgap NFAs

**Figure 10:**
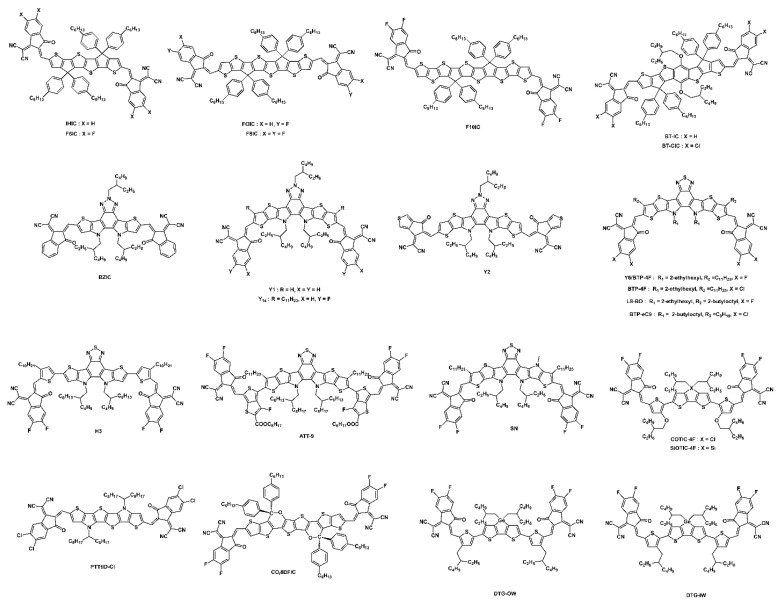
Molecular structures of fused CPDT, fused BDT, Y6, and other novel NFA.

The addition of an electron-rich oxygen atom into the backbone of a ladder-type donor core not only improves the electron-donating ability of the core but also improves the molecular planarity, effectively reducing the bandgap of NFAs. The chemical structures of fused CPDT, fused BDT, A-DA′D-A-type, and other novel narrow-bandgap NFAs are shown in Figure 10, and their optoelectrical properties and solar cell performance are summarized in Table 2.

In 2017, Xiao et al. modified the ladder-type carbon-bridged (IDT) core to a ladder-type carbon–oxygen-bridged (CO5) core through an intramolecular demethanolization cyclization approach. Due to the stronger electron-donating ability and increased planarity of CO5 over IDT, the CO5IC molecule showed an absorption red shift and lower bandgap compared to IDTIC-based NFA (E_g_^opt^ = 1.62 eV vs. 1.70 eV) [96]. Later, the same group reported a low-bandgap (E_g_^opt^ = 1.26 eV) NFA, CO*i*8DFIC. Owing to the enhanced planarity and good charge mobility, the OSC device based on PTB7-Th:CO*i*8DFIC achieved a PCE of 12.16%, with an outstanding Jsc of 26.12 mA cm^−2^ [97].

**Figure 11:**
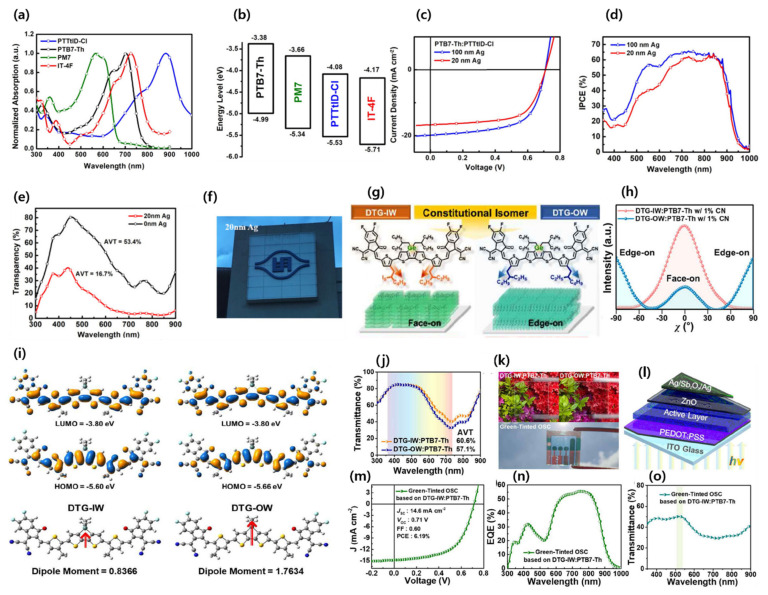
(**a**) Absorption spectra of PTTtID-Cl, PTB7-Th, PM7, and IT-4F in thin film; (**b**) energy levels of PTTtID-Cl, PTB7-Th, PM7, and IT-4F; (**c**) *J–V* characteristics; (**d**) IPCE spectra; (**e**) transparency diagrams of PTB7-Th:PTTtID-Cl-based devices with different thickness of Ag electrodes, and (**f**) digital picture of a semitransparent PTB7-Th:PTTtID-Cl-based device with 20 nm Ag. Reproduced with permission [100]. Copyright 2021, John Wiley and Sons. (**g**) Isomeric structures of DTG-IW and DTG-OW with face-on and edge-on orientation; (**h**) circular cut profiles of π–π (010) stacking for optimized blend film of DTG-IW/PTB7-Th and DTG-OW/PTB7-Th; (**i**) DFT calculated HOMO and LUMO orbitals and dipole moment vectors of DTG-IW and DTG-OW; (**j**) transmittance spectra of optimized DTG-IW/PTB7-Th and DTG-OW/PTB7-Th blend films; (**k**) Photograph of the active layers and full cell with illustration of green-tinted ST-OSC; (**l**) device architecture; (**m**) J*–V* characteristics; (**n**) EQE spectra, and (**o**) transmittance of the green-tinted ST-OSC based on DTG-IW/PTB7-Th. Reproduced with permission [102]. Copyright 2020, American Chemical Society.

In 2003, Rasmussen and co-workers developed a general synthetic route for the efficient preparation of N-functionalized coplanar and strong electron-donating dithieno[3,2-b:2′,3′-d]pyrroles (DTPs) [98]. In contrast to commonly fused aromatic hydrocarbons with a sp^3^-hybridized carbon bridge, the introduction of the nitrogen bridge with a sp^2^ hybridized lone pair of electrons could enhance the effective conjugation length and electron-donating ability and reduce the bandgap of DTP-based NFAs. The application of fused dimers of DTPs in organic conjugated materials showed high carrier mobilities with reduced bandgaps [99]. Wang et al. designed and synthesized an A-D-A-configured NFA, PTTtID-Cl, made of the fused dimer of a DTP donor core, which resulted in strong absorption with a lower bandgap than IT-4F (1.45 vs. 1.54 V, calculated by CV measurements) (Figure 11a–f). The ST-OSC device based on PTB7-Th:PTTtID-Cl achieved a PCE of 7.7% with an AVT of 16.7% [100].

**Table 2:** Optoelectronic properties and photovoltaic parameters of fused CPDT, fused BDT, Y6, and other novel NFA-based materials.

Device Structure ^a^	NFA	HOMO eV	LUMO eV	E_g_^opt^ eV	Voc V	Jsc mAcm^2^	FF %	PCE % (ST-OSC)	PCE % (OSC)	AVT %	LUE %	CRI	CIE 1931 (x, y)	Ref.
ITO/ZnO/PTB7-Th:IHIC/ MoO_3_/Au/Ag	IHIC	−5.45	−3.93	1.38	0.75	19.01	68	9.77	4.20	36	3.51	86	0.273, 0.309	[83]
ITO/ZnO/PTB7-Th:6TIC/MoO_3_/Al/Ag	6TIC	−5.21	−3.83	1.37	0.82	14.58	63	7.62	11.07	23.3	1.8	−	−	[84]
ITO/PEDOT:PSS/J61:BT-IC/PDINO/Al	BT-IC	−5.32	−3.85	1.43	0.87	16.35	67	−	9.56	−	−	−	−	[85]
ITO/PEDOT:PSS/J71:BTIC/PDINO/Al	BT-IC	−	−	−	0.90	17.75	66	−	10.46	−	−	−	−	[85]
ITO/ZnO/PTB7-Th: FOIC/MoO_3_/Au/Ag	FOIC	−5.36	−3.92	1.32	0.74	20.0	70	10.3	12.0	37.4	3.85			[86]
ITO/ZnO/PTB7-Th: ITIC3/MoO_3_/Ag	ITIC3	−5.54	−3.90	1.55	0.76	16.8	63	−	8.09	−	−	−	−	[86]
ITO/ZnO/PTB7-Th:F6IC/MoO_3_/Ag	F6IC	−5.66	−4.02	1.36	0.61	18.07	64	−	7.01	−	−	−	−	[87]
ITO/ZnO/PTB7-Th:F8IC/MoO_3_/Ag	F8IC	−5.43	−4.00	1.27	0.64	25.12	68	−	10.9	−	−	−	−	[87]
ITO/ZnO/PTB7-Th:F10IC/MoO_3_/Ag	F10IC	−5.26	−3.96	1.25	0.73	20.83	67	−	10.2	−	−	−	−	[87]
ITO/ZnO/PCE-10: BT-CIC/MoO_3_/Ag	BT-CIC	−5.49	−4.09	1.33	0.68	18.0	68	7.1	11.2	43	3.05	91	0.29, 0.32	[88]
ITO/PEDOT:PSS/HFQx-T:BZIC/PDINO/Al	BZIC	−5.42	−3.88	1.45	0.84	12.67	59	−	6.30	−	−	−	−	[89]
ITO/ZnO/PBDB-T:Y1/MoO_3_/Ag	Y1	−5.45	−3.95	1.44	0.87	21.69	71	−	13.3	−	−	−	−	[90]
ITO/ZnO/PBDB-T:Y2/MoO_3_/Ag	Y2	−5.43	−4.04	1.40	0.81	22.89	71	−	13.2	−	−	−	−	[90]
ITO/SnO_2_/PBDB-T:Y14/MoO_3_/Ag	Y14	−5.56	−4.01	1.30	0.79	22.48	71	12.67	14.67	23.69	3.00	−	−	[91]
ITO/PEDOT:PSS/PM6:Y6/PDINO/Al	Y6	−5.65	−4.10	1.33	0.83	25.3	75	−	15.7	−	−	−	−	[45]
ITO/ZnO/PM6:Y6/PEDOT:PSS	Y6	−5.7	−3.9	1.33	0.75	15.8	63	7.46		36.4	2.72	−	0.28, 0.31	[92]
ITO/PEDOT:PSS/PM6:Y6/PDIN/Au/Ag	Y6	−	−	−	0.85	20.35	71	12.37	15.83	18.6	2.30	−	0.255, 251	[93]
ITO/PEDOT:PSS/PTB7-Th:H3/ZnO/PEI/Ag/TeO_2_	H3	−5.40	−3.90	1.5 ^b^	0.2	17.30	68	8.38	12.35	50.09	4.06	77	0.291, 0.339	[94]
ITO/PEDOT:PSS/PTB7-Th:ATT-9/PDINN/Ag	ATT-9	−5.45	−3.90	1.15	0.66	20.7	69	9.37	13.35	35	3.33	−	−	[95]
ITO/ZnO/PBDB-TF:BTP-4F/MoO_3_/Al	BTP-4F	−5.65 ^c^	−4.02 ^c^	1.63 ^c^	0.83	24.9	75	−	15.3	−	−	−	−	[66]
ITO/ZnO/PBDB-TF:BTP-4F/MoO_3_/Al	BTP-4Cl	−5.68 ^c^	−4.12 ^c^	1.56 ^c^	0.86	25.4	75	−	16.1	−	−	−	−	[66]
ITO/ZnO/PTQ11:TPT10/MoO_3_/Ag	TPT10	−5.52	−3.99	1.36	0.88	24.79	74	−	16.32	−	−	−	−	[78]
ITO/ZnO/PTB7-Th:CO*i*8DFIC/MoO_3_/Ag	CO*_i_*8DFIC	−5.50	−3.88	1.26	0.68	26.12	68	−	12.16	−	−	−	−	[97]
ITO/ZnO/PTB7-Th:PTTtID-Cl/MoO_3_/Ag	PTTtID-Cl	−5.53	−4.08	1.45 ^b^	0.73	17.7	60	7.7	8.9	16.7	1.29	−	0.325, 0.306	[100]
ITO/ZnO/PTB7-Th:COTIC-4F/MoO_3_/Ag	COTIC-4F	−5.26	−4.16	1.10	0.56	20.3	56	−	7.4	−	−	−	−	[101]
ITO/ZnO/PTB7-Th:SiCOTIC-4F/MoO_3_/Ag	SiOTIC-4F	−5.28	−4.11	1.17	0.65	21.6	61	−	9.0	24.2	2.1	−	−	[101]
ITO/PEDOT:PSS/PTB7-Th/DTG-IW/ZnO/Ag/Sb_2_O_3_/Ag	DTG-IW	−5.63	−4.07	1.32	0.71	14.6	60	6.19	9.16	50.4	3.11	−	−	[102]
ITO/PEDOT:PSS/PTB7-Th:DTG-OW/ZnO/Al	DTG-OW	−5.67	−4.01	1.29	0.69	16.2	66	−	7.45	−	−	−	−	[102]

^a^ Active layer was highlighted in blue color and photovoltaic parameters (Voc, Jsc, and FF) as well as the device structure were shown for ST-OSC when present both opaque and semitransparent device efficiency. ^b^ Calculated from CV measurements. ^c^ Obtained from the square wave voltammetry (SWV) method.

Inspired by the promising characteristics, such as high coplanarity, strong electron-donating ability, and strong π–π stacking tendency, of CPDT, dithienogermole (DTG), and dithienosilole (DTS), Lee et al. designed and synthesized narrow-bandgap NFAs, COTIC-4F and SiOTIC-4F (1.10 and 1.17 eV, respectively). OSC devices with a PTB7-Th donor showed a PCE of 7.4 and 9.0% for COTIC-4F and SiOTIC-4F, respectively, indicating the potential of the tricyclic bithiophene system fused with the periodic table of group 14 (IVa) elements, like C, Si, and Ge [101]. Later, Yang’s group designed and reported two novel germaniums containing dithienogermole (DTG)-based narrow-bandgap isomeric (alkyl position inward vs. outward) NFAs, named DTG-IW and DTG-OW. The GIWAXS measurements showed highly confined face-on crystallites in the solid state of DTG-IW (inwards side chain) compared to DTG-OW (outward side chain) (Figure 11g–o). The OSC of the PTB7-Th:DTG-IW blend revealed a better PCE of 9.16% than for PTB7-Th:DTG-OW (PCE of 7.45%) due to the higher electron mobility of DTG-IW than DTG-OW. Further, the fabricated ST-OSC device achieved a PCE of 6.19% with an AVT of 50.4% by using a semitransparent Ag/Sb2O3/Ag electrode [102]. 

#### 3.1.5. Narrow-Bandgap Polymers

**Figure 12:**
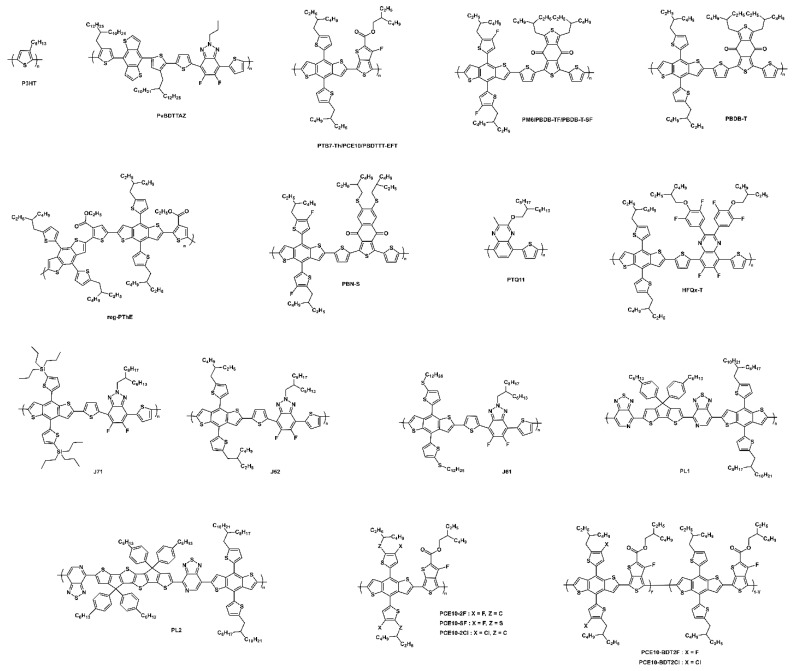
Chemical structures of all polymers described in this manuscript.

**Figure 13:**
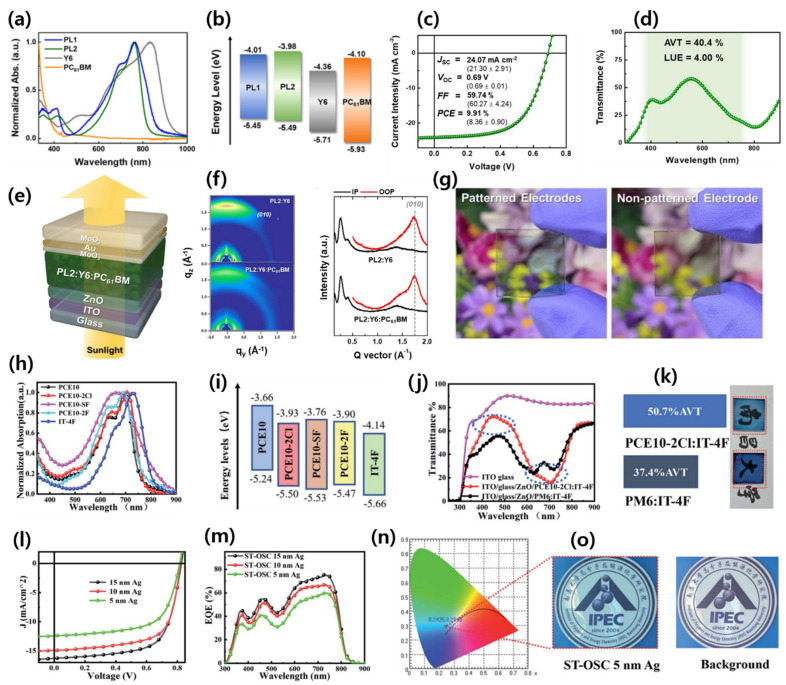
(**a**) Absorption spectra of PL1, PL2, Y6, and PC61BM thin films; (**b**) energy level diagrams of PL1, PL2, Y6, and PC_61_BM; (**c**) *J–V* curve, (**d**) transmittance spectrum, and (**e**) device architecture of the ST-OSC based on PL2 ternary blends; (**f**) GIWAXS images and line-cut profiles of the binary and ternary blend films of PL2 based polymer donor; and (**g**) photograph of real ST-OSC devices with the patterned electrodes and non-patterned electrode. Reproduced with permission [104]. Copyright 2023, Elsevier. (**h**) Normalized absorption spectra of PCE10, PCE10-2Cl, PCE10-SF, and PCE10-2F films; (**i**) energy levels of PCE10, PCE10-2Cl, PCE10-SF, PCE10-2F and IT-4F; (**j**) transmittance spectra of PM6:IT4F and the PCE10-2Cl:IT-4F active layer on the ITO glass; (**k**) AVT value and photograph of PM6:IT-4F and PCE10-2Cl:IT-4F active layer film on ITO glass with the same active layer thickness of 110 nm; (**l**) *J–V* curves; (**m**) EQE spectra; (**n**) color coordinates of ST-OSC device with thicknesses of 10 nm Ag, and (**o**) photographs of 5 nm Ag thickness ST-OSC and without device. Reproduced with permission [105]. Copyright 2021, Royal Society of Chemistry.

At present, narrow-bandgap polymer donors like PCE10 (also known as PTB7-Th) are widely used in high-performance ST-OSCs but, unfortunately, their high HOMO energy level and poor blend morphology with Y6 and its derivatives limit the Voc and FF of the device and, thus, PCE. Therefore, the rational design of polymers for active-layer blending is crucial to elevate the Voc, FF, and performance of ST-OSCs [103]. The chemical structures of all polymers described in this manuscript are shown in Figure 12. Recently, Lee and co-workers designed and synthesized NIR-absorbing polymer donors by using CPDT and a fused CPDT core for PL1 (E_g_ 1.44 eV) and PL2 (E_g_ 1.51 eV), respectively. Both polymers showed lower HOMO energy levels, strong absorption in the NIR region, and high transmittance in the visible region compared to PTB7-Th. The optimized ST-OSC device based on PL2:Y6:PC_61_BM demonstrated an efficient PCE of 9.91% and an AVT of 40.4%, with a remarkable LUE of 4.0% (Figure 13a–g) [104]. This work provided a significant molecular design strategy for novel narrow-bandgap polymer donors to further improve the ST-OSC performance and AVT simultaneously.

Chen and co-workers designed and reported a series of novel narrow-bandgap polymer with lower HOMO and LUMO energy levels by utilizing fluorine, chlorine, and sulfur functional atoms in the widely used polymer PCE10. The PCE10-2F, PCE10-SF, and PCE10-2Cl polymers showed enhanced Voc, and the device based on PCE10-2Cl:IT-4F achieved a PCE of 10.72% for OSC with a high Voc of 0.82 V. The ST-OSC device with PCE10-2Cl:IT-4F achieved an efficient PCE of 8.25 with an AVT of 33% and suggested that the rational design of polymers and combination with acceptors are very promising for increasing the PCE of ST-OSCs (Figure 13h–o) [105]. Later, the same group reported novel terpolymers, namely PCE10-BDT2F and PCE10-BDT2Cl. The designed terpolymers showed deeper HOMO energy levels, higher extinction coefficients, and enhanced compatibility with Y6 compared to PCE10. The fabricated ST-OSC device based on PCE10-BDT2F:Y6 achieved a PCE of 10.85%, AVT of 41.08%, and with an outstanding LUE of 4.46% [106]. Huang et al. reported a molecular-weight-regulated strategy for efficient sequential deposition to enhance the performance of ST-OSCs. The strategy significantly improved the morphology and charge dynamic and reduced the energy loss. The OSC device based on a narrow-bandgap polymer and Y6 acceptor (PCE10-2F:Y6) showed an outstanding PCE of 14.53% with a high Jsc of 26 mA cm^−2^ and FF of 70.32%. The resultant ST-OSC device revealed an efficient PCE of 10.01% and AVT of 50.05%, with an excellent LUE of 5.01% without complex optical engineering [107].

#### 3.1.6. Multicomponent Strategy

In the past few years, the active layers developed with the multicomponent strategy (ternary and quaternary) have been confirmed to further enhance the performance of OSCs. In contrast to binary blends, ternary/quaternary systems offer distinct advantages, such as broadened and intensified the optical absorption, effective charge separation, transport, and extraction, as well as improved device stability [108,109,110].

**Table 3:** Optoelectronic properties and photovoltaic parameters of novel narrow-bandgap polymers and other multicomponent-based materials.

Device Structure ^a^	Polymer/NFA	HOMO eV	LUMO eV	E_g_^opt^ eV	Voc V	Jsc mAcm^2^	FF %	PCE % (ST-OSC)	PCE % (OSC)	AVT %	LUE %	CRI	CIE 1931 (x, y)	Ref.
ITO/ZnO/PL1:Y6:PC_61_BM/MoO_3_/Ag	PL1	−5.45	−4.01	1.44	0.66	20.41	56	−	7.54	−	−	−	−	[104]
ITO/ZnO/PL2:Y6:PC_61_BM/MoO3/Au/MoO_3_	PL2	−5.49	−3.98	1.51	0.69	24.07	60	9.91	11.62	40.4	4.0		−	[104]
ITO/ZnO/IT-4F:PCE10-2F/MoO_3_/Ag	PCE10-2F	−5.47	−3.90	1.57	0.79	11.12	43	−	3.71	−	−	−	−	[105]
ITO/ZnO/IT-4F:PCE10-2F/MoO_3_/Ag	PCE10-SF	−5.53	−3.76	1.70	0.68	8.48	42	−	2.40	−	−	−	−	[105]
ITO/ZnO/IT-4F:PCE10-2F/MoO_3_/Ag	PCE10-2Cl	−5.50	−3.93	1.57	0.82	16.96	68	8.25	10.72	33	2.72	69	0.235, 0.287	[105]
ITO/PEDOT:PSS/PCE10-BDT2F:Y6/PDINO/Ag	PCE10-BDT2F	−5.42	−3.83	1.59 ^b^	0.75	20.73	70	10.85	13.80	41.08	4.46	79	−	[106]
ITO/PEDOT:PSS/PCE10-BDT2Cl:Y6/PDINO/Ag	PCE10-BDT2Cl	−5.41	−3.81	1.60 ^b^	0.74	28.85	66	−	12.09	−	−	−	−	[106]
ITO/ PEDOT:PSS/PCE10-2F/Y6/PDINO/Ag/MoO_3_	PCE10-2F	−5.47	−3.87	1.57	0.78	17.79	71	10.01	14.53	50.05	5.01	−	0.265, 0.290	[107]
ITO/ZnO/PTB7-Th:PTTtID-Cl:IT4F/MoO_3_/Ag	PTTtID-Cl:IT-4F	−	−	−	0.85	17.5	69	9.1	12.0	−	−	−	0.276, 0.213	[100]
ITO/PEDOT:PSS/PBDB-TF:L8-BO/Bis-FIMG/Ag	L8-BO	−5.68	−3.90	1.78 ^b^	0.86	22.99	79	15.61	18.25	8.98	1.4	−	−	[111]
ITO/PEDOT:PSS/PBDB-TF:L8-BO:BTP-eC9/Bis-FIMG/Ag	BTP-eC9:L8-BO ^c^	−	−	−	0.85	19.00	79	12.95	19.35	38.67	5.0	−	−	[111]
ITO/PEDOT:PSS/PM6:SN/PDINN/Ag	SN	−5.51	−3.82	1.40	0.82	21.78	68	12.2	14.3	22	2.68	−	−	[112]
ITO/PEDOT:PSS/PM6:Y6:SN/PDINN/Ag	Y6:SN	−	−	−	0.82	22.98	74	14.0	17.5	20.2	2.83	95	0.283, 0.283	[112]
ITO/PEDOT:PSS/PM6: BTP-eC9/PDINN/Ag	BTP-eC9	−5.67	−4.05	1.62 ^b^	0.75	18.73	76	10.5	17.81	29.27	3.07	80	0.280, 0.239	[113]
(LiF/TeO_2_)4/ITO/PEDOT:PSS/PM6:BTP-eC9:L8-BO/PDINN/Ag/(LiF/TeO_2_)8/LiF	BTP-eC9:L8BO	−	−	−	0.95	17.97	75	11.44	18.24	57.5	5.35	85	0.305, 0.336	[113]
ITO/PEDOT:PSS/PCE10-2F:PM6:Y6/PDINO-3/Ag/MnO_3_	PCE10-2F:PM6	−	−	−	0.78	21.99	71	12.25	16.77	36.57	4.48	−	0.283, 0.348	[114]

^a^ Active layer was highlighted in blue color and photovoltaic parameters (Voc, Jsc, and FF) as well as the device structure were shown for ST-OSC when present both opaque and semitransparent device efficiency. ^b^ Calculated from CV measurements.

**Figure 14:**
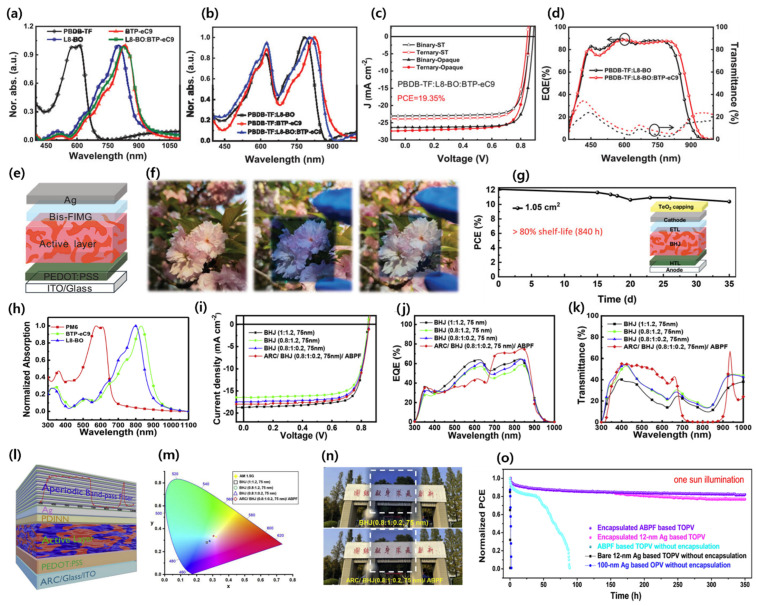
(**a**) Absorption spectra of PBDB-TF, L8-BO, BTP-eC9, and L8-BO:BTP-eC9 films; (**b**) absorption spectra of the binary and ternary blend films; (**c**) *J–V* curves of the PBDB-TF:L8-BO and PBDB-TF:L8-BO:BTP-eC9 opaque devices and the corresponding ST-OSCs; (**d**) EQE spectra of PBDB-TF:L8-BO and PBDB-TF:L8-BO:BTP-eC9 opaque OSCs and the transmittance spectra of the corresponding ST-OSCs; (**e**) device architecture of the OSC; (**f**) surrounding images of without ST-OSC device (left), with ST-OSC and 0 nm TeO_2_ (middle), and with ST-OSC and 45 nm TeO_2_ optical modulation (right), and (**g**) stability of the large area (1.05 cm^2^) unencapsulated ST-OSC stored in a N2-filled glove box. Taken from [111]. Copyright 2021, John Wiley and Sons. (**h**) Normalized film absorption of PM6, BTP-eC9, and L8-BO; (**i**) *J–V* curves, (**j**) EQE curves, and (**k**) transmittance spectra of ST-OSCs with and without aperiodic band-pass filter (ABPF, aperiodic LiF/tellurium dioxide [TeO_2_]]/LiF); (**l**) ABPF-integrated ST-OSCs device architecture; (**m**) CIE 1931 color coordinates of ST-OSCs; (**n**) outdoor photographs of ST-OSCs with and without ABPF; (**o**) PCE change with maximum power point tracking test time under one sun illumination (LED lamp) in the ambient air condition. Reproduced with permission [113]. Copyright 2023, Elsevier.

Chen and co-workers efficiently converted NIR light while allowing for the transmission of visible light for high-performance ST-OSCs. The ST-OPV based on binary blend PBDB-TF:L8-BO achieved a PCE of 15.61%, AVT of 8.98, and LUE of 1.4%. The carrier dynamics and selective absorption were successfully assessed for different donor/acceptor ratios and the inclusion of a third component (ternary strategy). The ternary device based on PBDB-TF:BTP-eC9:L8-BO achieved an outstanding PCE of 12.95%, AVT of 38.67%, and significantly improved LUE of 5.0%. Furthermore, a large-area ST-OSC device (1.05 cm^2^) showed a record high PCE of over 12%, demonstrating its ability for upscaling (Figure 14a–g) [111]. Asymmetric acceptors can achieve a balance between solubility and crystallinity, in addition to potential alterations in conformation, affecting molecular packing. These molecules show a higher dipole moment and stronger binding energy, which can enhance the intermolecular interactions and, thus, improve the device performance. Liu et al. designed and reported asymmetric NFA and SN by N-substituting in a central fused core, which showed absorption of 40 nm red shifted compared to Y6. Interestingly, SN exhibited a very low nonradiative voltage loss of only 0.15 eV, which is lower than monocrystalline silicon cells (0.18 eV). The low nonradiative voltage loss was attributed to a higher photoluminescence quantum yield (PLQY), increased dipole moments, and lower HOMO energy offset of SN than Y6. Due to higher absorption in the NIR region and aforementioned characteristics, the ST-OSC device based on PM6:SN achieved a PCE of 12.2% with an AVT of 22%, which is 4% higher in the LUE than PM:Y6 device. Benefiting from the absorption extension in the NIR region by blending the red-shifted acceptor, the group fabricated a ternary device based on PM6:Y6:SN and achieved a PCE of 14%, with an AVT of 20% [112].

To achieve a LUE over 5%, the materials involved in the active layer must have absorption in the selective NIR and/or UV region while allowing for transmittance in the visible region to secure a high Jsc and AVT. Liu et al. fabricated an ST-OSC with PM6: BTP-eC9 (D:A = 0.8:1.2) active layer thickness of 75 nm and achieved a PCE of 10.5%, AVT of 29.27%, and LUE of 3.07%. A ternary strategy was utilized by adding L8BO to boost the performance. Compared with the binary blend, the ternary device exhibited an improved LUE from 3.07% to 3.31%. Further, an antireflection coating (ARC) integrated with a glass substrate device structure was utilized to improve the visible transmittance, resulting in an efficient LUE of 5.35%, with an enhancement in the AVT of 57.5% (Figure 14h–o) [113]. Chen’s group reported a facile strategy to improve the PCE and AVT simultaneously by employing highly photovoltaic materials with highly transparent materials. Due to synergistic complementary absorption, improved morphology, and efficient charge dynamics, the PCE of the ternary device based on the PCE10-2F:PM6:Y6 (0.7:0.3:2.5 *w*/*w*%) achieved a high PCE of 12.25%, with an outstanding LUE of 4.48% compared to the binary blend (PCE 11.07% and LUE 4.27%) [114].

## 4. Conclusions and Outlook

In OSCs, one of the primary concerns is stability. Moisture, oxygen, and light all contribute to the degradation of organic materials and result in significant reductions in efficiency and lifetime. The development of stable organic molecules is crucial for achieving long-term stability. Another challenge is scalability. Despite the fact that organic molecules have flexibility in molecular design and control properties, producing high-quality materials on a larger scale is still difficult. Organic solar cells are commercially viable because of their cost-effectiveness. The synthesis of high-performance OSC materials often involves several steps and complex purification techniques. Finding facile approaches to cost-effective material synthesis is crucial for realizing the full potential of OSCs. Over the past few years, opaque and semitransparent OSC technologies have made immense progress, opening a new paradigm for solar markets. The state-of-the-art efficiency for opaque OSCs and ST-OSCs has been increased to over 19% and 11% (AVT of ~57%), respectively. Also, there has been promising progress in the stability of these fascinating technologies, and some reports are expected to operate for several years while retaining at least 80% of their initial efficiency. In addition, efficient OSCs do not contain poisonous elements like lead (Pb), as most high-efficiency perovskite solar cells contain this. In terms of optical properties, OSCs are extremely versatile, making them a great choice for emerging applications, like smart power-generating windows, wearable devices, automobiles, and agriculture. Interestingly, for particular applications, such as building windows, electronic displays, and sensors, the AVT can be further compromised by wavelength-selective materials and device engineering. Nevertheless, achieving a satisfactory balance between PCE and AVT of ST-OSCs remains a great challenge. Material design, optimization of the active layer morphology, development of transparent electrodes, device engineering, and ensuring device stability all demand careful attention to facilitate the practical application of multifunctional ST-OSCs.

The advent of new semiconducting NFAs is the main driving force for the fast development of ST-OSCs. In this critical review, we focused on summarizing the recent progress in the development of different classes of highly competent photoabsorbent NIR materials for efficient ST-OSCs. The example molecules used in this study clearly illustrate the direct link between molecular design and the performance of ST-OSCs. Therefore, based on the aforementioned discussions, here, we will propose several approaches and help researchers further utilize the potential of ST-OSCs.

According to the molecular orbital theory, the optoelectronic properties, including the optical bandgap, energy levels, and stability of NFAs, can be effectively tuned by controlling the ICT interaction between the electron donor and acceptor moiety. In ST-OSCs, narrow-bandgap semiconductors, which harvest photons at NIR wavelengths and do not affect transparency in the visible region, will remain a preferred route to satisfy strict absorption conditions. To further narrow the bandgap, increasing the electron-donating/accepting ability of the donor/acceptor, extending the π-conjugation with a multifunctional group, which additionally improves π-π stacking and charge-transporting behavior, and maximizing the molar absorptivity are crucial to realizing high PCE and AVT. Also, as discussed in this manuscript, most of the NFA structures were synthesized using electron-deficient acceptor end units, IC-2F, or IC-2Cl, and there is plenty of space to explore new efficient acceptor end groups for ultra-narrow-bandgap materials for efficient ST-OSCs. Recently, asymmetric NFA structures have also proven to be excellent strategies for additional benefits due to their high dipole moment and intermolecular binding energy. Therefore, further utilization of such types of novel NFAs could enhance the performance of ST-OSCs.

In terms of photocurrent generation, state-of-the-art NFA-based OSCs are comparable to their perovskite and inorganic counterparts but lacking in Voc due to high voltage loss. In order to further enhance the Voc, the development of strong photoluminescence NFAs with efficient charge generation is important. Hybridization of the local exciton (LE) and charge transfer (CT) state can also promote luminescence and, thus, suppress the nonradiative recombination. However, due to the lack of understanding of NFA molecular structure relationships with their emissive properties, further efforts are needed to guide the design of highly emissive molecular materials. 

To date, the PTB7-Th polymer electron donor is widely used in efficient ST-OSCs, but due to its strong absorption in the 500–700 nm range, obstacles in the transmittance of the device have continued, and new electron-donor materials must be designed, with absorption compatibility, matching energy levels, balance charge transport properties, and compatibility of blend film morphology with NFAs. A novel ultra-wide bandgap with a deep HOMO energy level compatible with NIR NFA can achieve high AVT. The arrangement of molecules within active layers significantly affects the photon-harvesting ability and phase separation. Employing a multicomponent strategy and various treatments, such as solvent additives, thermal and vapor annealing, donor-acceptor ratios, and active layer thickness, offers an efficient means to adjust the molecular arrangement and phase separation within these layers. Also, approaching long-term solutions can circumvent the short exciton diffusion length (10–20 nm) through diverse strategies, like increasing the extent of crystalline order, tailoring molecular packing arrangements, and molecular templating. Practicing the potential of the singlet fission phenomenon (two free electrons per absorbed photon) in a systematic way to double the photocurrent could be a game changer in ST-OSCs. 

To achieve efficient ST-OSCs, developing transparent electrodes with ultra-low sheet resistance, appropriate energy alignment, high electrical conductivity and transmittance in the visible spectrum, and high reflectance in the NIR region is required. Along with active-layer materials and electrodes, exploring suitable interface materials with characteristics, such as low-temperature processing, low trap states, high conductivity, fine work function, and good ohmic contact between the active layer and electrodes, needs to be considered. Light, oxygen, moisture, and heat all affect the stability of organic solar cells, so these basic problems need to be figured out for practical applications. To enter the niche market, the large-area fabrication of ST-OSCs must achieve a balance between cost, PCE, AVT, and stability. Theoretical calculations are also very important in evaluating molecular properties. The emergence of artificial intelligence (AI) technology is anticipated to speed up material discovery, screening, optimization, and the implementation of processing conditions and parameters. We strongly believe that, once the current challenges are collectively tackled through innovative material design, device engineering, and technological advancement, ST-OSCs will massively improve quality of life. We hope that our review article further encourages research in this direction.
